# Towards understanding non-equivalence of α and β subunits within human hemoglobin in conformational relaxation and molecular oxygen rebinding†

**DOI:** 10.1039/d1sc00712b

**Published:** 2021-04-15

**Authors:** Sergei V. Lepeshkevich, Igor V. Sazanovich, Marina V. Parkhats, Syargey N. Gilevich, Boris M. Dzhagarov

**Affiliations:** B. I. Stepanov Institute of Physics, National Academy of Sciences of Belarus 68 Nezavisimosti Ave Minsk 220072 Belarus s.lepeshkevich@ifanbel.bas-net.by; Central Laser Facility, Research Complex at Harwell, STFC Rutherford Appleton Laboratory Harwell Campus OX11 0QX UK igor.sazanovich@stfc.ac.uk; Institute of Bioorganic Chemistry, National Academy of Sciences of Belarus 5 Academician V. F. Kuprevich Street Minsk 220141 Belarus

## Abstract

Picosecond to millisecond laser time-resolved transient absorption spectroscopy was used to study molecular oxygen (O_2_) rebinding and conformational relaxation following O_2_ photodissociation in the α and β subunits within human hemoglobin in the quaternary R-like structure. Oxy-cyanomet valency hybrids, α_2_(Fe^2+^–O_2_)β_2_(Fe^3+^–CN) and α_2_(Fe^3+^–CN)β_2_(Fe^2+^–O_2_), were used as models for oxygenated R-state hemoglobin. An extended kinetic model for geminate O_2_ rebinding in the ferrous hemoglobin subunits, ligand migration between the primary and secondary docking site(s), and nonexponential tertiary relaxation within the R quaternary structure, was introduced and discussed. Significant functional non-equivalence of the α and β subunits in both the geminate O_2_ rebinding and concomitant structural relaxation was revealed. For the β subunits, the rate constant for the geminate O_2_ rebinding to the unrelaxed tertiary structure and the tertiary transition rate were found to be greater than the corresponding values for the α subunits. The conformational relaxation following the O_2_ photodissociation in the α and β subunits was found to decrease the rate constant for the geminate O_2_ rebinding, this effect being more than one order of magnitude greater for the β subunits than for the α subunits. Evidence was provided for the modulation of the O_2_ rebinding to the individual α and β subunits within human hemoglobin in the R-state structure by the intrinsic heme reactivity through a change in proximal constraints upon the relaxation of the tertiary structure on a picosecond to microsecond time scale. Our results demonstrate that, for native R-state oxyhemoglobin, O_2_ rebinding properties and spectral changes following the O_2_ photodissociation can be adequately described as the sum of those for the α and β subunits within the valency hybrids. The isolated β chains (hemoglobin H) show similar behavior to the β subunits within the valency hybrids and can be used as a model for the β subunits within the R-state oxyhemoglobin. At the same time, the isolated α chains behave differently to the α subunits within the valency hybrids.

## Abstract 2

O_2_ rebinding and conformational relaxation following O_2_ photodissociation were studied on picosecond to millisecond time scale in the α and β subunits within human hemoglobin in the quaternary R-like structure.

## Graphical abstract

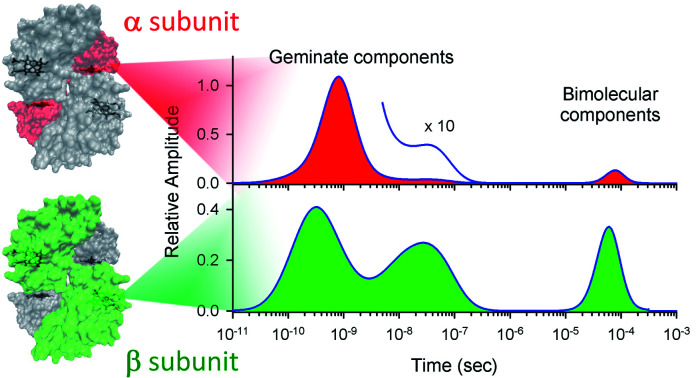

## 1 Introduction

**Fig. 1:**
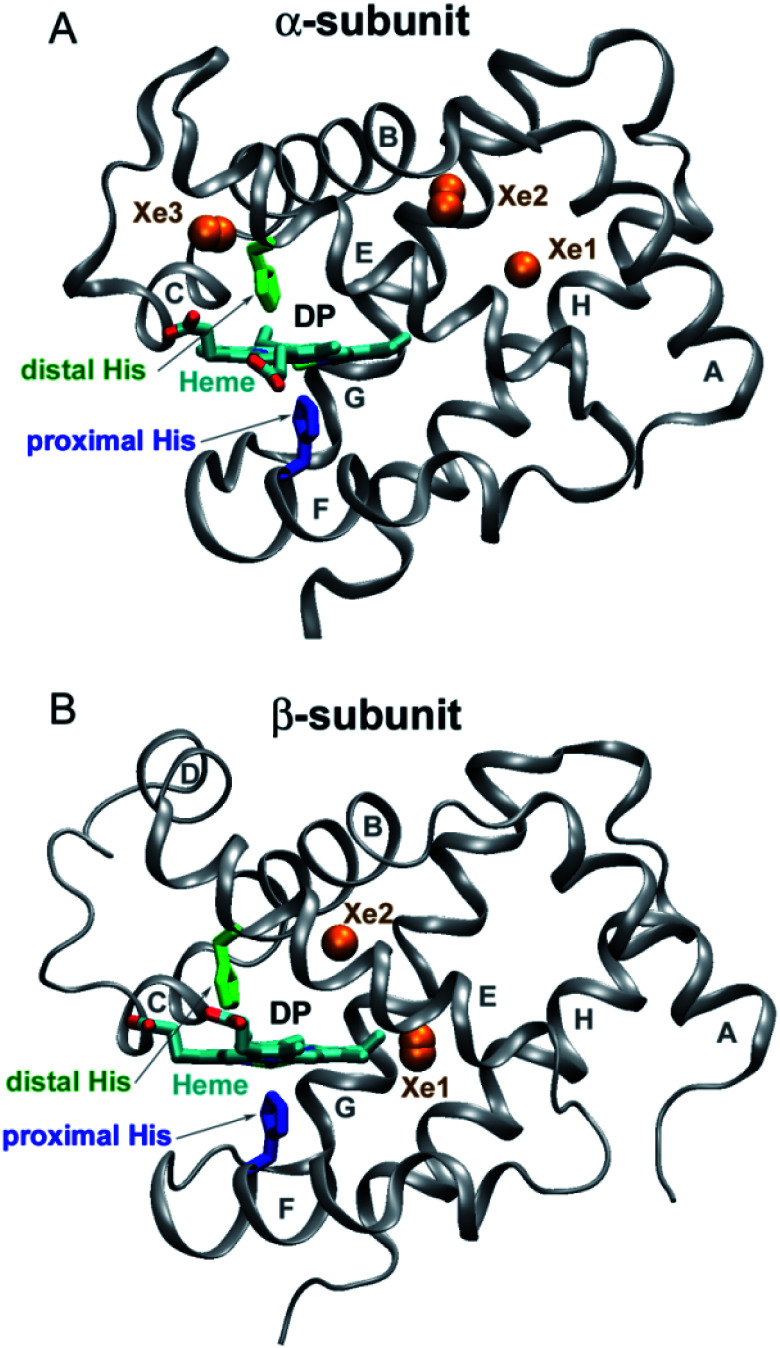
Structure of the α and β subunits of human Hb (panel (A) and (B), respectively) (PDB entry 2w6v).^18^ The distal heme pocket (primary docking site) is labeled as DP. The distal and proximal histidines are labeled in green and blue, respectively. Xe sites are represented by labeled orange spheres.

Human hemoglobin (Hb) is an allosteric protein that binds and transports molecular oxygen (O_2_). Human Hb is a tetramer composed of two α and two β subunits (Fig. 1). Each subunit contains one identical heme group (an iron-protoporphyrin IX), in which the iron ion is bound to the proximal histidine (His). The ferrous heme iron reversibly binds diatomic ligands such as O_2_ and CO on the opposite side of the proximal His. Human Hb binds four ligands cooperatively. As tetrameric Hb is liganded, its quaternary structure changes and the ligand affinity increases. The deoxygenated Hb has a low-affinity quaternary structure (T-state) and the liganded one has a high-affinity structure (R-state). Human Hb continues to serve as an excellent model for studying nonlinear and cooperative interactions in multisubunit proteins.^1^ Despite a large amount of work so far, there is no definitive agreement on how ligand-induced conformational changes influence individual ligand binding properties of the α and β subunits in the different conformational forms of tetrameric Hb. This information is necessary to understand the molecular mechanism of cooperative oxygenation of human Hb as well as the mechanism of O_2_ transport and tissue oxygenation.

Taking advantage of the photosensitivity of the chemical bond between the ferrous heme iron and its sixth ligand,^2^ laser time-resolved spectroscopy has been used for kinetic studies of ligand binding^3–11^ and conformational changes^12,13^ following ligand photodissociation in human Hb. Using ultrafast transient absorption measurements, it has been determined that a photoproduct with a deoxy-like absorption spectrum appears within 350 fs after laser excitation.^14^ After photodissociation, the ligand remains temporarily trapped in the distal heme pocket of the Hb subunits (Fig. 1).^15^ Subsequently, the ligand can rebind to the heme iron or migrate through the protein matrix into the solvent, from where it can return into the protein. Ligand rebinding is readily modulated by steric hindrance in the distal heme pocket.^3^ Moreover, the intrinsic heme reactivity is controlled through a change in proximal constraints during conformational relaxation following the ligand photodissociation.^16^ In turn, ligand migration is modulated by structural determinants such as internal cavities and hydrogen-bonding residues in the distal heme pocket.^3,17^ Small internal cavities are generally hydrophobic and have an ability to bind Xe atoms by non-covalent specific interactions.^18^ Previous crystallographic analysis of the low affinity deoxygenated T-state Hb, filled with Xe atoms under pressure, has identified the positions of Xe binding cavities in both the α and β subunits (Fig. 1).^18^ It has been suggested that these internal cavities serve as transient areas for ligands migrating through the protein matrix in both Hb subunits.

Most of the flash photolysis studies of human oxyhemoglobin have dealt with the rebinding of O_2_ from the solvent (the bimolecular O_2_ rebinding), which occurs on a microsecond to millisecond time scale at ambient temperature.^3–7,19^ The O_2_ rebinding from within the protein matrix (the geminate O_2_ rebinding) has been investigated using nanosecond,^8,9^ picosecond,^10,11^ and femtosecond time resolved spectroscopy.^20^ A complete kinetic description of the O_2_ rebinding with triliganded R-state Hb at room temperature has been presented.^21,22^ These studies reported triple-exponential geminate O_2_ rebinding in which photolyzed population rebinds *via* two prompt geminate phases with 0.14 and 1 ns time constants, and *via* another delayed geminate phase with a ∼30 ns time constant. Assigning all these geminate phases to specific processes in specific subunits is a very difficult task. At present, there is some controversy in the literature regarding the assignment of the geminate phases to specific subunits.^15,21^ The main problem with the interpretation is that optical absorption spectra of hemes in the α and β subunits of human Hb are almost identical, so that it is not possible to study the behavior of the individual subunits. A variety of experimental approaches has been developed to solve the problem of functional differences between the α and β subunits within human Hb. Among them are (i) measurements of the ligand binding properties of the isolated α and β chains,^8^ (ii) selective chemical modification or mutation of key amino acid residues,^7^ (iii) X-ray crystallography of photolyzed liganded Hbs,^15,23^ and (iv) construction of metal^5,9^ and valency^24^ hybrid Hbs in which only one type of subunits is capable to bind O_2_.

The principal aim of this study was to determine how ligand-induced conformational changes influence the individual O_2_ rebinding properties of the α and β subunits in the R-state Hb. To do that we used oxy-cyanomet valency hybrids α_2_(Fe^2+^–O_2_)β_2_(Fe^3+^–CN) and α_2_(Fe^3+^–CN)β_2_(Fe^2+^–O_2_) as models for the oxygenated R-state Hb. These valency hybrids possess several important properties. First, only the ferrous subunits within these hybrids reversibly bind O_2_, while the ferric subunits do not. Second, under aerobic conditions used in the present study, no valency exchange occurs between the oxyheme and cyanometheme sites.^25^ Thus, no noticeable electron transfer is expected to occur in the study. Third, based on extensive NMR^26^ and X-ray structural analysis of liganded human Hbs (see references in ref. 27 and 28), it can be concluded that the solution structure of oxy-cyanomet valency hybrids of human Hb under low-salt conditions is a quaternary R-like structure, which is similar to those of ferrous oxy and carbonmonoxy human Hb. It should be mentioned that, under low-salt conditions, both cyanomet and normal oxyhemoglobin have similar thermodynamic stability.^29^ Under these conditions, cyanomet human Hb is crystallized with a quaternary R-like structure similar to that observed in carbonmonoxy human Hb.^27^ Therefore, the oxy-cyanomet valency hybrids, α_2_(Fe^2+^–O_2_)β_2_(Fe^3+^–CN) and α_2_(Fe^3+^–CN)β_2_(Fe^2+^–O_2_), are extremely useful to investigate the O_2_ rebinding in the individual α(Fe^2+^) and β(Fe^2+^) subunits within Hb in the R-state. In the present work, we used a single time-resolved transient absorption spectrometer^30^ to study the geminate O_2_ rebinding as well as conformational relaxation following the O_2_ photodissociation in the α and β subunits within the Hb valency hybrids on the entire picosecond to millisecond time scale.

## 2 Experimental

### 2.1 Preparation of isolated hemoglobin chains

Human Hb was purified from freshly drawn blood. The isolated α and β chains were subsequently obtained by the *p*-mercuribenzoate (PMB) method^31^ with minor modifications. The isolated chains with bound PMB (α^PMB^ and β^PMB^ chains) were separated in either the oxy or the carbonmonoxy form (see ESI† for details). The isolated α and β chains were regenerated to their –SH forms (α^SH^ and β^SH^ chains) using dithiothreitol (DTT)^32^ (see ESI† for details).

### 2.2 Preparation of hemoglobin valency hybrids

The Hb valency hybrids of the type α_2_(Fe^2+^–O_2_)β_2_(Fe^3+^–CN) and α_2_(Fe^3+^–CN)β_2_(Fe^2+^–O_2_) were prepared by mixing the isolated chains with free –SH groups in the oxy form with their partner chains in the cyanomet form^33^ (see ESI† for details). The cyanomet forms of the isolated α^SH^ and β^SH^ chains were obtained from the carbonmonoxy-derivatives by oxidation with potassium ferricyanide in the presence of sodium cyanide (see ESI† for details). The procedure for the formation of the cyanomet chains was additionally applied for the preparation of the fully ligated cyanomet Hb, α_2_(Fe^3+^–CN)β_2_(Fe^3+^–CN). All the spectroscopy experiments were performed in 50 mM Tris–HCl buffer, pH 8.2, at 19 °C. Concentration of the Hb valency hybrids, the oxy- and cyanomet Hb as well as the isolated Hb chains used in the present study was in the range of 130 to 200 μM in heme. The protein samples were checked before and after the experiment by UV-vis spectroscopy.

### 2.3 Time-resolved spectroscopy

Picosecond to millisecond time-resolved spectra in the visible (Soret) region were measured on the ULTRA^30^ apparatus in the Time-Resolved Multiple Probe Spectroscopy mode^34^ at the Central Laser Facility (STFC Rutherford Appleton Laboratories, Harwell, UK) (see ESI† for details). The time-resolved spectrometer^30^ used here allows to detect transient absorption spectra in the Soret region (from about 430 to 460 nm) in the time range from 1 ps up to 800 μs after the laser photoexcitation with a sensitivity of 10^−5^ absorbance units. The degree of photolysis in all the experiments did not exceed 5%.

## 3 Results and discussion

### 3.1 Thermal relaxation and cyanide photodissociation

After laser photoexcitation of oxygen- and cyanide-bound heme proteins, a large amount of energy is deposited into the heme, raising its temperature.^35^ As the energy is transferred to protein and water, the heme cools down. The thermal relaxation is nonexponential in time. In particular, the thermal relaxation in myoglobin has been previously characterized by two phases: an initial fast cooling process (a few ps time constant) and a slower one (∼20 ps time constant).^36,37^ Recently, a prompt subpicosecond phase has been detected.^38^ In the cyanide-bound heme proteins, photodissociation of cyanide from the ferric subunits is additionally expected. Previously,^39^ photodissociation of cyanide from ferric myoglobin and horseradish peroxidase has been observed after laser excitation at 413 nm. The subsequent geminate CN rebinding has been demonstrated to proceed on the picosecond time scale with a rate ≈(3.6 ps^−1^), the efficiency of CN escape from the protein matrix being ∼10^−4^. It is well known that the absorption spectra of heme proteins in the visible region are very sensitive to both temperature and ligand binding. Therefore, taking into account the above-mentioned processes, the time-resolved transient absorption spectra of studied Hb valency hybrids are expected to exhibit contributions of thermal relaxation and cyanide photodissociation on the tens of picoseconds time scale.

To determine the time window of the thermal relaxation and cyanide rebinding which are expected to occur in the present study, we measured the time-resolved spectra following photolysis of the oxy- and cyanomet Hb and its isolated chains as well as the oxy-cyanomet valency hybrids of human Hb in the time range from 1 ps up to 800 μs (not shown). Using singular value decomposition (SVD),^40^ it was found that the time-resolved spectra exhibit contributions of the thermal relaxation and, possibly, cyanide rebinding over times shorter than 40 ps but exhibit only contributions of the O_2_ rebinding and concomitant protein conformational changes thereafter. Because we are only interested in the O_2_ rebinding and concomitant protein conformational changes, we will hereafter analyze the time-resolved spectra measured at time delays of 40 ps and longer.

### 3.2 Time-resolved transient absorption spectra

**Fig. 2:**
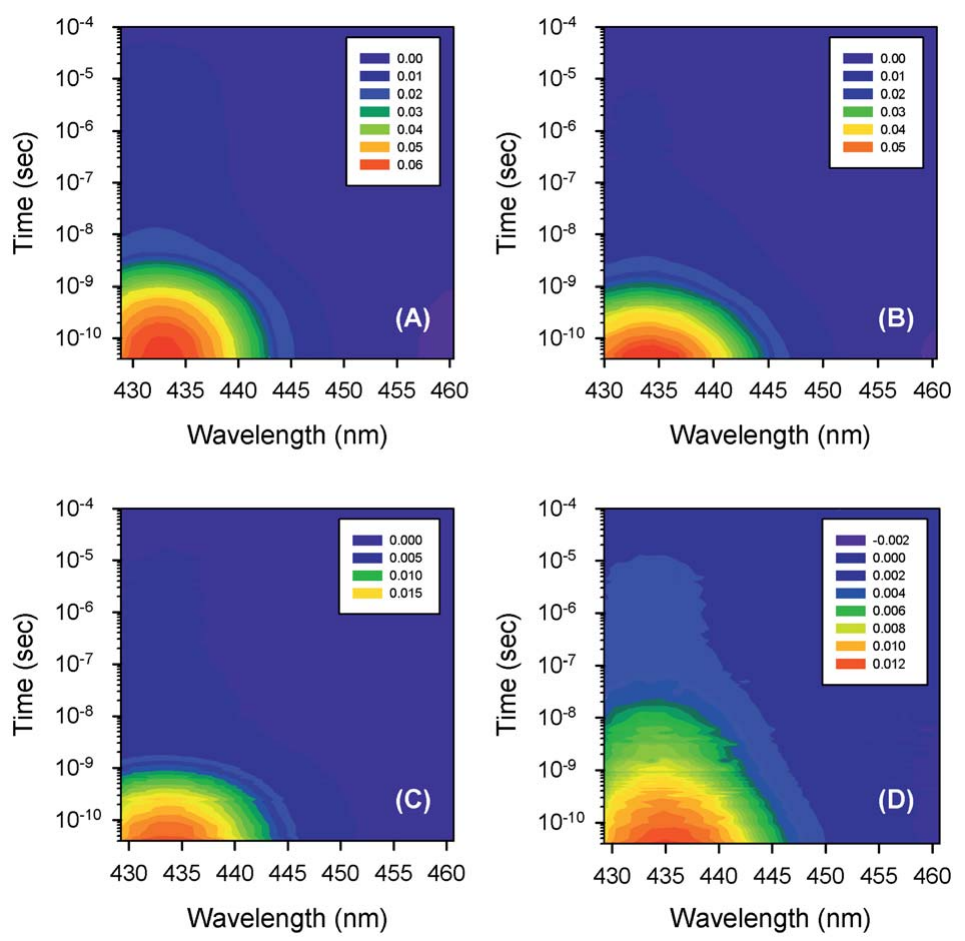
Contour plots of the time-resolved absorption difference spectra measured after O_2_ photodissociation from (A) the isolated α^SH^ chains, (B) oxyhemoglobin, (C) the α subunits within α_2_(Fe^2+^–O_2_)β_2_(Fe^3+^–CN) valency hybrids, and (D) the β subunits within α_2_(Fe^3+^–CN)β_2_(Fe^2+^–O_2_) valency hybrids. The time-resolved spectra, obtained for the isolated β^SH^ chains, are similar to those for the β subunits within α_2_(Fe^3+^–CN)β_2_(Fe^2+^–O_2_) valency hybrids (D) and are not shown in the figure. Conditions: 50 mM Tris–HCl buffer, pH 8.2, at 19 °C. Protein concentrations are in the range of 130 to 200 μM in heme. Excitation wavelength, *λ*_exc_ = 543 nm.

**Fig. 3:**
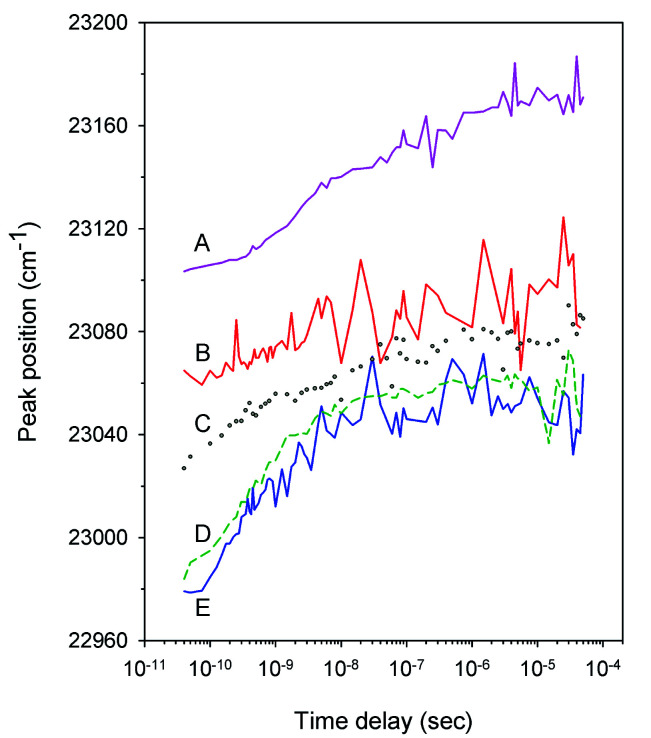
Time dependence of the position of the positive peak near 434 nm in the transient spectra *D*(*λ*,*t*) following O_2_ photodissociation from (A) the isolated α^SH^ chains (pink, solid), (B) the α subunits within α_2_(Fe^2+^–O_2_)β_2_(Fe^3+^–CN) valency hybrids (red, solid), (C) oxyhemoglobin (gray, circles), (D) the isolated β^SH^ chains (green, short dash), and (E) the β subunits within α_2_(Fe^3+^–CN)β_2_(Fe^2+^–O_2_) valency hybrids (blue, solid).

Typical time-resolved transient absorption spectra, *D*(*λ*,*t*), following photolysis of the O_2_ complexes of the heme proteins are shown as contour plots in Fig. 2. A closer inspection of the present transient spectra reveals a shift of the positive peak near 434 nm (∼23 040 cm^−1^) from low to high energy as time progresses (Fig. 3). The ultimate magnitude of this spectral shift was calculated as Δ*ν* = *ν*(∞) − *ν*(0), where *ν*(∞) is the peak position at a time which is long enough compared to the time scale of the shift, *ν*(0) is the peak position just after the thermal relaxation. The magnitude of Δ*ν* as well as the peak positions *ν*(0) and *ν*(∞) are reported in ESI Table S1.† The magnitude of Δ*ν* ranges from 37 ± 14 cm^−1^ for α_2_(Fe^2+^–O_2_)β_2_(Fe^3+^–CN) valency hybrid to 70 ± 9 cm^−1^ for α_2_(Fe^3+^–CN)β_2_(Fe^2+^–O_2_) valency hybrid. The magnitude of Δ*ν* as well as the peak positions *ν*(0) and *ν*(∞) for the native oxyhemoglobin are similar to those obtained by averaging the corresponding values for the two valency hybrids.

**Fig. 4:**
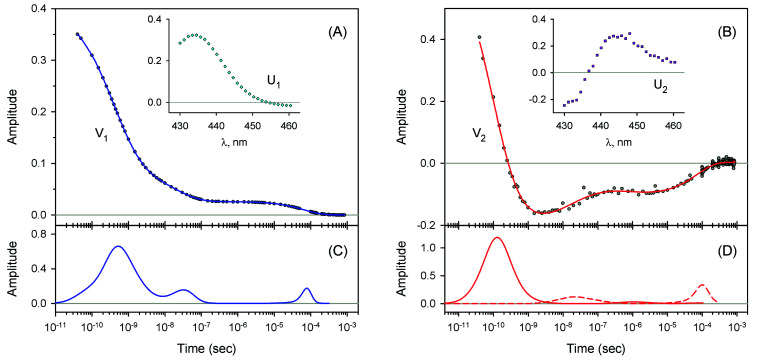
Singular value decomposition of the time-resolved spectra *D*(*λ*,*t*) measured after O_2_ photodissociation from native oxyhemoglobin. (A) Time-dependent amplitudes (V_1_) of the first basis spectrum. (B) Time-dependent amplitudes (V_2_) of the second basis spectrum. In panel (A) and (B), the time-dependent amplitudes and their fit obtained with the MEM analysis are reported as circles and solid lines, respectively. The first and the second basis spectra, U_1_ and U_2_, are shown in the insets to panels (A) and (B), respectively. (C) Lifetime distribution derived by MEM from V_1_. (D) Two lifetime distributions derived by MEM from V_2_. The two lifetime distributions, corresponding to decaying and rising kinetics, are presented as solid and dash lines, respectively.

**Fig. 5:**
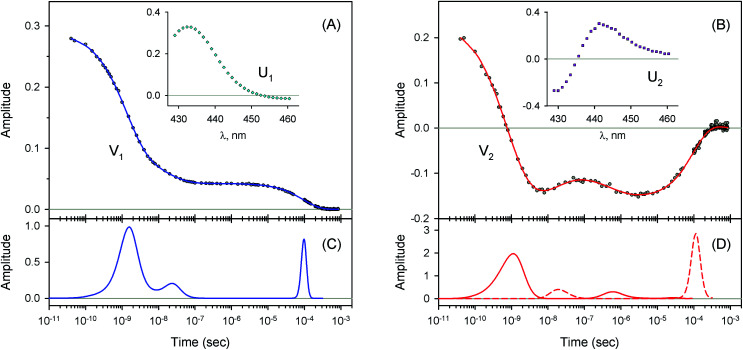
Singular value decomposition of the time-resolved spectra *D*(*λ*,*t*) measured after O_2_ photodissociation from the isolated α^SH^ chains. Description of each panel and symbols used is the same as for Fig. 4.

**Fig. 6:**
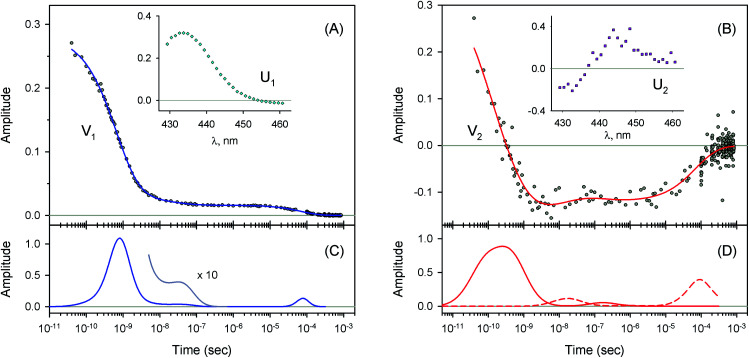
Singular value decomposition of the time-resolved spectra *D*(*λ*,*t*) measured after O_2_ photodissociation from the α subunits within α_2_(Fe^2+^–O_2_)β_2_(Fe^3+^–CN) valency hybrids. Description of each panel and symbols used is the same as for Fig. 4. In panel (C), to visualize the smallest peak (local maximum) in the lifetime distribution, a part of the distribution in the region from 5 × 10^−9^ s to ∼10^−6^ s is zoomed in.

**Fig. 7:**
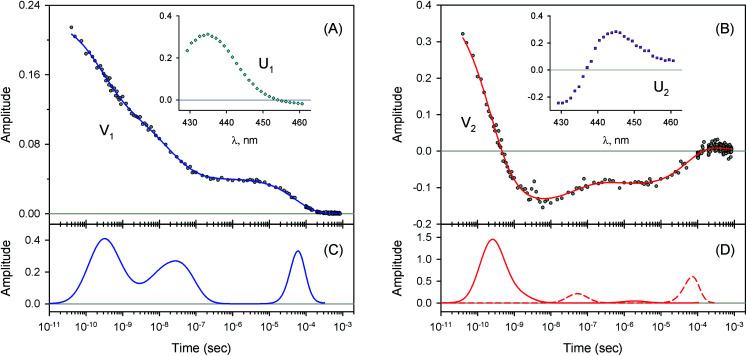
Singular value decomposition of the time-resolved spectra *D*(*λ*,*t*) measured after O_2_ photodissociation from the β subunits within α_2_(Fe^3+^–CN)β_2_(Fe^2+^–O_2_) valency hybrids. Description of each panel and symbols used is the same as for Fig. 4.

The measured transient absorption spectra at different time delays, *D*(*λ*,*t*), can be viewed as the columns of a matrix D. In order to describe time courses of the observed spectral changes, we used SVD of the data matrix D for each heme protein (see ESI† for details). All singular values, obtained for every studied protein, are presented in ESI Fig. S1.† It was found that the first two orthonormal basis spectra, U_1_ and U_2_, and their corresponding time-dependent amplitudes (orthonormal kinetic vectors), V_1_ and V_2_, form the best two-component representation of the data matrix D in the least-square approach. A data matrix, **D̃**, re-created from these two SVD components, provides the experimental data set to be modeled in the subsequent analysis (see Section 3.5). The results of SVD analysis for the heme proteins are shown on Fig. 4–7 and ESI Fig. S2.†

The first two basis spectra, U_1_ and U_2_, with their corresponding time-dependent amplitudes, V_1_ and V_2_, which make the main contribution to the observed photoinduced absorption changes, are shown in panel (A) and (B) of these Figures. The first basis spectrum, U_1_, represents a deoxy minus oxy difference spectrum averaged over all times. Hence, the temporal evolution of the first amplitude vector, V_1_, is an excellent approximation to the ligand rebinding curve. In turn, the second basis spectrum, U_2_, represents a deviation from the average spectrum and describes spectral changes in the region of the deoxy Soret band at ∼430 nm. The time course of the second amplitude vector, V_2_, is determined by (i) an overall decrease in amplitude due to the ligand rebinding and (ii) spectral changes of the remaining deoxyhemes caused by kinetic hole burning and protein conformational relaxation.^12^ The obtained time-dependent amplitudes, V_1_ and V_2_, were subsequently subjected to the maximum entropy method (MEM) analysis^41^ which extracts model-independent lifetime distributions from the kinetics (see ESI† for details). One or two distributions of the effective log-lifetimes, *g*(log *τ*) and *h*(log *τ*), were extracted from the data. The fit *F*_*i*_ to datum *D*_*i*_ at time *t*_*i*_ can be written as1
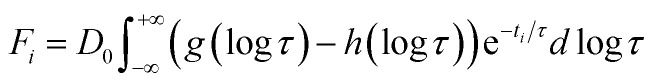
where *D*_0_ is a normalization constant, *g*(log *τ*) and *h*(log *τ*) are the lifetime distributions that correspond to decaying and rising kinetics, respectively. The extracted lifetime distributions are displayed in panel (C) and (D) of Fig. 4–7 and ESI Fig. S2.† For each appreciable peak in every recovered lifetime distribution, the area, *a*, and the mean, *τ*, were determined according to eqn (7) and (8) from ref. 42, respectively. In the cases when only one distribution is extracted from the data, the fractional contribution of each peak, *F*, to the total peaks area was calculated. The MEM-derived parameters characterizing the lifetime distributions, extracted from the time-dependent amplitudes, V_1_ and V_2_, are presented in Tables 1 and 2, respectively.

### 3.3 Molecular oxygen rebinding

For every studied heme protein, the MEM analysis of the first amplitude vector, V_1_, derived one lifetime distribution, which is characterized by three bands (Fig. 4C–7C and ESI Fig. S2C†). The first two bands that appear between 10^−11^ and 10^−6^ s are associated with the geminate O_2_ rebinding. The third band that appears between 10^−5^ and 10^−3^ s is associated with the bimolecular O_2_ rebinding. In Table 1, the fractional contributions 
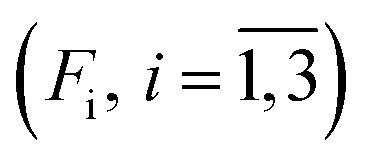
 of all three MEM peaks characterizing the O_2_ rebinding to the heme proteins are presented. All the fractional contributions are normalized to unity. In this case, *F*_1_ and *F*_2_ determine the fraction for the faster and slower geminate phase, respectively. The sum of *F*_1_ and *F*_2_ is equal to the fraction of geminate O_2_ rebinding, *F*_gem_. In turn, *F*_3_ determines the fraction of bimolecular O_2_ rebinding, which is equal to the efficiency of O_2_ escape from the protein matrix after photodissociation, *δ*_escape_.^22,43^ Additionally, in Table 1, the values of *δ*_escape_, determined previously for the isolated α^SH^ and β^SH^ chains,^43^ for native oxyhemoglobin^22^ as well as for the α and β subunits within native oxyhemoglobin,^22^ are summarized for comparison. As it is seen from Table 1, the values of *F*_3_, evaluated in the present work for the isolated α^SH^ and β^SH^ chains as well as for the native oxyhemoglobin, are in a good agreement with the corresponding values of *δ*_escape_ determined previously.^22,43^ Moreover, the values of *F*_3_, obtained in the present study for the α and β subunits within the Hb valency hybrids, are in excellent agreement with the values of *δ*_escape_ obtained for the corresponding α and β subunits within native oxyhemoglobin in the R-state.^22^ These results support the idea that the Hb valency hybrids are good models to elucidate the inequivalence of the α and β subunits within human Hb in the R-state with respect to the O_2_ rebinding.

**Table 1:** MEM-derived parameters characterizing lifetime distributions obtained for V_1_^a^

Protein	*τ*_1_ (ns)	*τ*_2_ (ns)	*τ*_3_ (μs)	*F*_1_ (× 10^−2^)	*F*_2_ (× 10^−2^)	*F*_3_ (× 10^−2^)	*δ*_escape_ (× 10^−2^)
αO_2_	1.37	23	96	72	13	15	20 ± 4^b^
βO_2_	0.32	31	66	57	28	15	21 ± 4^b^
Native HbO_2_	0.48	30	68	81	12	7	9.7 ± 1.6^c^
α_2_(Fe^2+^–O_2_)β_2_(Fe^3+^–CN)	0.76	44	75	91.5	2.8	5.7	
α_2_(Fe^3+^–CN)β_2_(Fe^2+^–O_2_)	0.34	23	58	47	35	18	
α within native HbO_2_							4.8 ± 1.0^c^
β within native HbO_2_							15 ± 3^c^

a Here, *τ*_*i*_
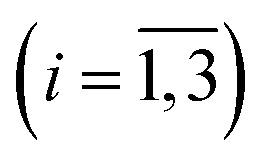
 is the mean of the *i*th MEM peak; *F*_*i*_
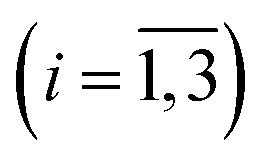
 is the fractional contribution of the *i*th MEM peak to the total peaks area; *δ*_escape_ is the efficiency of ligand escape from the protein after photodissociation. The uncertainties are presented as 95% confidence intervals. In the present study, protein concentrations were 130 to 200 μM in heme, 50 mM Tris–HCl buffer, pH 8.2, at 19 °C.

b Data from ref. 43, protein concentration was 100 μM in heme, 50 mM Tris–HCl buffer, pH 8.2, at 21 °C.

c Data from ref. 22, protein concentration was 100 μM in heme, 10 mM Tris–HCl buffer, pH 8.5, at 21 °C.

As follows from our data, the α and β subunits within the tetrameric valency hybrids exhibit different geminate O_2_ rebinding properties (Fig. 6C and 7C, respectively). The α subunits within α_2_(Fe^2+^–O_2_)β_2_(Fe^3+^–CN) valency hybrids show one dominant rapid geminate phase with a lifetime distribution peak at 0.76 ns and the largest fractional contribution being 0.915 (Table 1, *τ*_1_ and *F*_1_). The fraction of the slower scarcely observed second geminate component with the peak at 44 ns was found to be equal to 0.028 (Table 1, *τ*_2_ and *F*_2_). In contrast to the α subunits, the β subunits within α_2_(Fe^3+^–CN)β_2_(Fe^2+^–O_2_) valency hybrids show two distinct geminate phases with similar fractional contributions of 0.47 and 0.35 (Table 1, *F*_1_ and *F*_2_). It should be noted that it is the β subunits within the tetrameric valency hybrids that have the fastest geminate phase peaked at 0.34 ns, the second geminate component being peaked at 23 ns (Table 1, *τ*_1_ and *τ*_2_). Taking into account that the primary quantum yield of O_2_ photodissociation, *γ*_0_,^43^ is the same (0.23 ± 0.03) for tetrameric oxyhemoglobin and its isolated α and β chains,^11^ we consider that *γ*_0_ for the α and β subunits within the valency hybrids is the same too. (The same value, within the experimental error, 0.28 ± 0.06, is observed for oxymyoglobin.^44^) Subsequently, it can be concluded that the dominant rapid geminate component (the lifetime distribution peak at 0.76 ns) observed for the α subunits as well as the two distinct geminate phases with similar fractional contributions (the lifetime distribution peaks at 0.34 and 23 ns) observed for the β subunits provide the major contribution to the total kinetic of the geminate O_2_ rebinding to human Hb in the R-state, the fastest geminate phase corresponding to the β subunits. The obtained α/β difference in the geminate O_2_ rebinding agrees well with that in the geminate CO rebinding reported previously by Anfinrud and co-workers.^15^

At the used protein concentrations, the isolated α^SH^ chains are predominantly monomers being in monomer/dimer equilibrium, whereas the isolated β^SH^ chains self-associate to form homo-tetramers, called hemoglobin H.^45^ In turn, the native Hb as well as the Hb valency hybrids are mainly tetramers. The α subunits were found to be sensitive to whether they are in the isolated monomeric state or incorporated into the tetrameric valency hybrids. The isolated α^SH^ chains (Fig. 5C) and the α subunits within tetrameric α_2_(Fe^2+^–O_2_)β_2_(Fe^3+^–CN) valency hybrids (Fig. 6C) have different O_2_ rebinding properties. Upon isolation of the α subunits, the fraction for the slower geminate phase, *F*_2_, increases by a factor of ∼5 at the expense of the dominant faster geminate phase (Table 1), and the lifetime distribution value, *τ*_1_, associated with the faster component increases from 0.76 to 1.37 ns. The values of *F*_i_ and *τ*_i_ for the β subunits within α_2_(Fe^3+^–CN)β_2_(Fe^2+^–O_2_) valency hybrids resemble those for the isolated β^SH^ chains (Table 1).

### 3.4 Conformational relaxation

For every studied heme protein, the MEM analysis of the second amplitude vector, V_2_, derived two lifetime distributions corresponding to decaying and rising kinetics (Table 2, Fig. 4D–7D and ESI Fig. S2D†). In these two lifetime distributions four kinetic components are observed in total. Three of them (two fastest components, *τ*_1_ and *τ*_2_, and the slowest one, *τ*_4_) occur to be in the same range as those observed in the lifetime distributions for the first amplitude vector, V_1_, whose temporal evolution is an approximation of the ligand rebinding curve (Table 1, Fig. 4C–7C and ESI Fig. S2C†). A distinctive feature of the lifetime distributions derived from V_2_ is a kinetic component *τ*_3_, which varied in the range from 0.18 to 1.82 μs (Table 2). The first three MEM peaks derived from V_2_ are expected to be caused not only by an overall decrease in amplitude due to the ligand rebinding but also due to conformational relaxation following ligand photodissociation (*vide infra*). It should be mentioned that conformational relaxation in heme proteins following ligand photodissociation has been extensively investigated by resonance Raman spectroscopy.^46–50^ In those experiments, protein conformational relaxation involving the iron-proximal histidine linkage (Fe–His) was followed by detecting temporal changes in the frequency of iron-histidine out-of-plane mode, *ν*(Fe–His). The Fe–His bond is a sole chemical link between the heme and globin and is very important for regulation of ligand binding.^51^ After ligand photodissociation, the heme structure changes from the planar to the domed one within 1 ps.^52,53^ A series of subsequent changes in *ν*(Fe–His) frequency occurs step-wise in the picosecond to microsecond time range.^46–50^ Temporal changes in the *ν*(Fe–His) frequency have been fitted using exponential functions. Published time constants of temporal changes in the *ν*(Fe–His) frequency for Hb,^46,48–50^ its hybrids,^47,50^ and isolated chains^46,48^ following the ligand photodissociation are collected in ESI Table S2.† A rather good correlation is observed between those time constants and the present data, namely, the means of the lifetime distribution peaks extracted from the second amplitude vector, V_2_ (ESI Fig. S3†). For comparison, in Table S2,† we also show the lifetime distribution data obtained for V_2_ in the current work. Taking into account the obtained correlation (Fig. S3 and Table S2†), it can be concluded that the spectral changes, observed in the present experiment in the region of the deoxy Soret band, are caused, at least in part, by structural changes of the heme accompanied by the Fe–His bond relaxation following the ligand photodissociation.

**Table 2:** MEM-derived parameters characterizing lifetime distributions obtained for V_2_^a^

Protein	*τ*_1_ (decay, ns)	*τ*_2_ (rise, ns)	*τ*_3_ (decay, μs)	*τ*_4_ (rise, μs)	*a*_1_	*a*_2_	*a*_3_	*a*_4_
αO_2_	0.82	21	0.69	111	1.65	−0.24	0.22	−0.88
βO_2_	0.29	50	1.15	90	1.33	−0.136	0.030	−0.37
Native HbO_2_	0.13	26	1.12	78	1.58	−0.196	0.041	−0.24
α_2_(Fe^2+^–O_2_)β_2_(Fe^3+^–CN)	0.20	17	0.18	76	1.31	−0.112	0.049	−0.31
α_2_(Fe^3+^–CN)β_2_(Fe^2+^–O_2_)	0.30	50	1.82	64	1.26	−0.136	0.033	−0.30

a Here, *τ*_*i*_ and a_*i*_
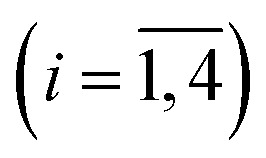
 are the mean and the area of the *i*th MEM peak, respectively. Positive and negative area values correspond to decaying and rising kinetics, respectively. Conditions: 50 mM Tris–HCl buffer, pH8.2, at 19 °C. Protein concentrations were in the range of 130 to 200 μM in heme.

The above-mentioned changes in the *ν*(Fe–His) frequency have been associated with changes in the tertiary structure.^46–50^ In particular, heme doming leads to structural rearrangements of the heme pocket in Hb and its isolated chains in ∼300 ps (Table S2,†*τ*_1_).^46^ In tens of nanoseconds (Table S2,†*τ*_2_), these structural changes are followed by the displacement of the distal E helix toward the heme plane, driven by motion of the proximal F helix in response to the relaxation of the Fe–His bond.^48–50^ In the case of tetrameric Hb in the R-state, a subsequent structural rearrangement within the β subunits induces the first step in the R–T transition at ∼3 μs (Table S2,†*τ*_3_) during which the αβ dimers rotate and establish the T “hinge” contacts between the β1 C helix and α2 FG corner.^47^ In turn, a structural rearrangement within the α subunits of Hb in the R-state is slower than that within the β subunits. Namely, the structural rearrangement within the α subunits induces the final step in the R–T transition at ∼20 μs (Table S2,†*τ*_4_) during which the α subunits rotate into their position, establishing the T “switch” contacts between the α2 C helix and the β1 FG corner.^47^ A time constant describing the final step in the R–T transition observed by resonance Raman scattering, ∼20 μs (Table S2,†*τ*_4_), has no corresponding time constant in the lifetime distributions extracted by us from the second amplitude vector, V_2_. This is due to the fact that, in the present experiment, the photoexcitation level was chosen to be sufficiently low to ensure that only single O_2_ ligand (statistically) is released by each tetrameric Hb molecule and the protein remains in the R-state.^54^

### 3.5 Kinetic models

**Scheme 1:**
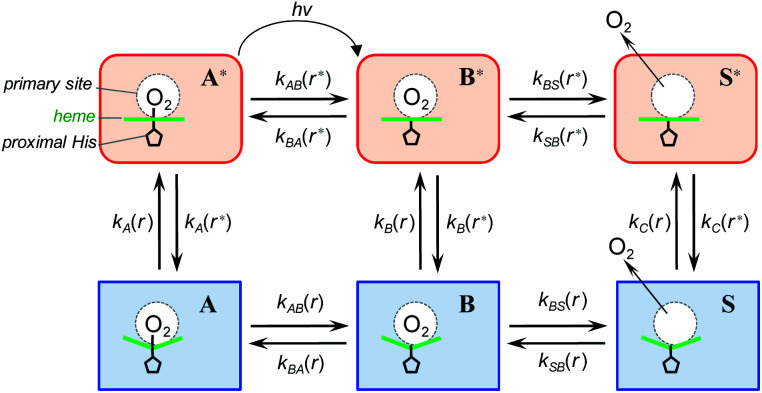
Basic kinetic model containing minimal set of tertiary/ligation states required to describe tertiary conformational changes as well as geminate and bimolecular O_2_ rebinding in ferrous Hb subunits.^12^

**Scheme 2:**
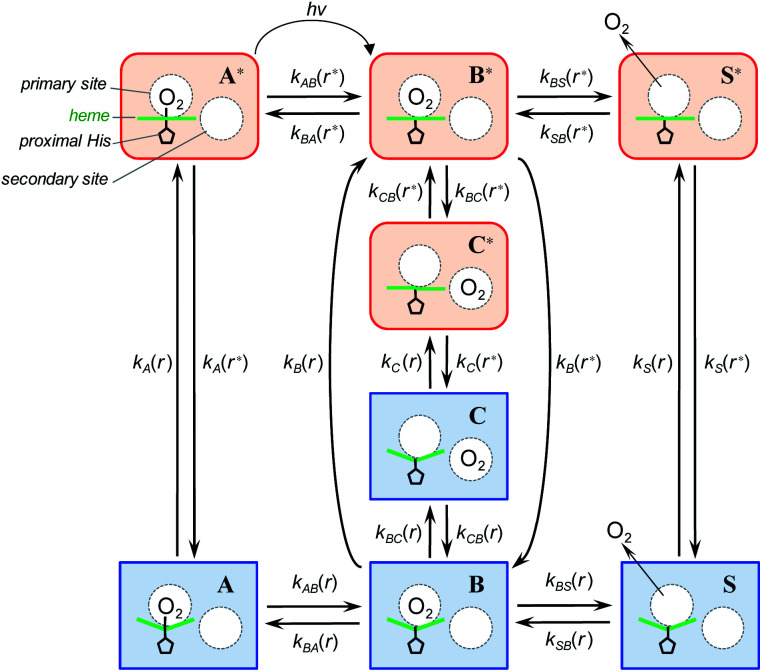
Extended kinetic model for tertiary conformational changes and O_2_ rebinding in ferrous Hb subunits, containing O_2_ migration between the primary and remote secondary docking site(s).

To describe the picosecond to millisecond kinetics of the O_2_ rebinding and concomitant conformational changes in the heme proteins, we followed the kinetic model proposed by Henry *et al.*^12^ This basic model (Scheme 1) contains a minimal set of states required to describe nonexponential conformational relaxation as well as the geminate and bimolecular O_2_ rebinding in ferrous Hb subunits. To account for the observed absorption changes induced by the O_2_ photodissociation, unliganded ferrous subunits are allowed to exist in two tertiary structures. The more rapidly rebinding species are designated as r*, while the more slowly rebinding ones (however possessing more stable tertiary structure) are designated as *r*. Oxygenated subunits in both tertiary states are assumed to have the same spectrum, while spectra of unliganded subunits in the r* and *r* tertiary structure are different. To describe the O_2_ rebinding, each tertiary state is proposed to exist in three possible ligation states (Scheme 1): O_2_ bound (A* or A), O_2_ inside the protein but not bound to the heme (B* or B), and ligand outside the protein (S* or S). Moreover, to account for the O_2_ migration between the primary docking site and possible remote (secondary) docking site(s), the basic kinetic model (Scheme 1) was extended by us to the one (Scheme 2) containing four ligation states. In the extended model (Scheme 2), the fourth additional ligation state C* or C in Scheme 2 corresponds to the state in which the O_2_ molecule is docked into a remote secondary docking site(s) accessible from the primary docking site (the distal heme pocket). The existence of discrete docking sites for the photodissociated CO within human Hb has been demonstrated previously by nanosecond laser flash photolysis studies, which found biphasic geminate CO rebinding to R-state Hb encapsulated in wet silica gels.^55,56^

In both models (Schemes 1 and 2), all liganded subunits prior to the photolysis are assumed to be in A* state, so that the ligand photodissociation initially populates exclusively B* state with the dissociated O_2_ ligand in the primary docking site of the affected subunit in the unrelaxed conformation, r*. In this state, subunits may undergo a conformational relaxation (B* → B), the ligand may geminately rebind (B* → A*) or escape into the solvent (B* → S*). In turn, in B state with the dissociated O_2_ ligand in the primary docking site of the affected subunit in the relaxed conformation, *r*, subunits may also undergo a conformational relaxation (B → B*), the ligand may geminately rebind (B → A) or escape into the solvent (B → S). Ligand from the solvent may enter any of the ligand-free subunits (S* → B* or S → B). Additionally, in the extended kinetic model (Scheme 2), the ligand may migrate between the primary and secondary docking site(s) of the affected subunits in both the unrelaxed r* (B* → C* or C* → B*) and relaxed *r* conformation (B → C or C → B). In both models (Schemes 1 and 2), the rate constant for the thermal dissociation A* → B* (Schemes, *k*_AB_(r*)) is considered to be negligibly small compared with the photodissociation rate. Additionally, the conformational relaxation A → A* (Schemes, *k*_A_(*r*)) is assumed to be instantaneous and irreversible, so that the rates for the thermal dissociation A → B (Schemes, *k*_AB_(*r*)) and the conformational change A* → A (Schemes, *k*_A_(r*)) play no role in fitting the kinetic data. The rate constants for O_2_ escape from and entry into the protein (*k*_BS_ and *k*_SB_, respectively) are assumed to be identical for the two tertiary subunit conformations r* and *r*. Moreover, it is assumed that the subunit conformation does not depend on the ligand position in the protein structure, so that the rates for the conformational relaxations B* → B, C* → C, and S* → S (marked on Schemes as *k*_B_(r*), *k*_C_(r*), and *k*_S_(r*), respectively) are identical, as are the reverse rates for the conformational changes B → B*, C → C*, and S → S* (marked on Schemes as *k*_B_(*r*), *k*_C_(*r*), and *k*_S_(*r*), respectively). Additionally, in the extended kinetic model (Scheme 2), the rate constants for the O_2_ migration between the primary and remote docking site(s) (*k*_BC_ and *k*_CB_) are assumed to be insensitive to the tertiary structure.

According to the mechanism of Hagen and Eaton,^57^ during the conformational relaxation, the system is not at the thermal equilibrium with respect to conformational substates of either r* or *r* tertiary state. To produce effects of interconversion of conformational substates occurring simultaneously with the change in the average conformation, labeled r* and *r*, time dependence has been introduced^12^ into the rate coefficients describing the interconversion between the two average conformations, r* and *r*:2
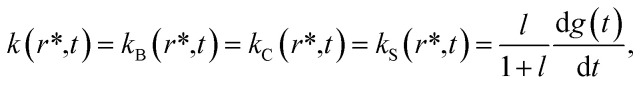
3
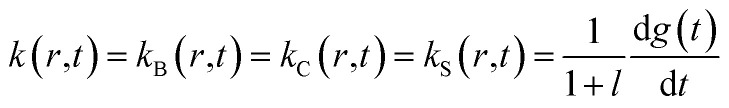
where *l* = *r*(∞)/r*(∞) is the equilibrium constant between the two tertiary states at *t* = ∞; the relaxation function *g*(*t*) was chosen to have the stretched exponential form,^58^

4 *g*(*t*) = (*kt*)^*β*^

with 0 < *β* < 1 and *k* = *k*(r* → *r*) + *k*(*r* → r*),^12^ where *k*(r* → *r*) and *k*(*r* → r*) are the rate constants for the interconversion between the r* and *r* tertiary states at equilibrium. Using these rate constants, the above-mentioned equilibrium constant *l* can be expressed as: *l* = *k*(r* → *r*)/*k*(*r* → r*).

At any given time after photodissociation, there is a non-equilibrium distribution among both the substates of r* and *r*,^12^ the individual substates having different geminate rebinding rate constants.^58^ Consequently, during the conformational relaxation at any given time, each unliganded tertiary species, r* and *r*, cannot be characterized by a single geminate rate. Rather than using the populations of r* and *r* with their individual geminate rate constants *k*_BA_(r*) and *k*_BA_(*r*) (Schemes 1 and 2), the same time-dependent geminate rebinding rate coefficient *k*_BA_(*t*) was employed^12^ for both tertiary species:

5 *k*_BA_(*t*) = *k*_BA_(r*,*t*) = *k*_BA_(*r*,*t*) = *k*_gem_(r*) (*k*_gem_(r*)/*k*_gem_(*r*))^*x*(*t*)−1^

where

6 *x*(*t*) = exp(−*g*(*t*))

describes the progress of the tertiary relaxation using the above-mentioned relaxation function *g*(*t*) (eqn (4)). In eqn (5), *k*_gem_(r*) represents the rate constant for the geminate O_2_ rebinding to ferrous subunits in the r* tertiary state before the beginning of the conformational relaxation. In turn, *k*_gem_(*r*) is the rate constant for the geminate O_2_ rebinding to subunits in the *r* tertiary state after the completion of the relaxation. In the present work we performed modeling the data matrix **D̃** so that to produce a set of time-dependent species populations and a set of corresponding species spectra (see ESI† for details). The adjustable parameters used in the modeling are summarized in ESI Table S3.† The resulting parameters for the basic and extended model are listed in Tables 3 and 4, respectively.

### 3.6 Time-dependent populations of unliganded states

**Fig. 8:**
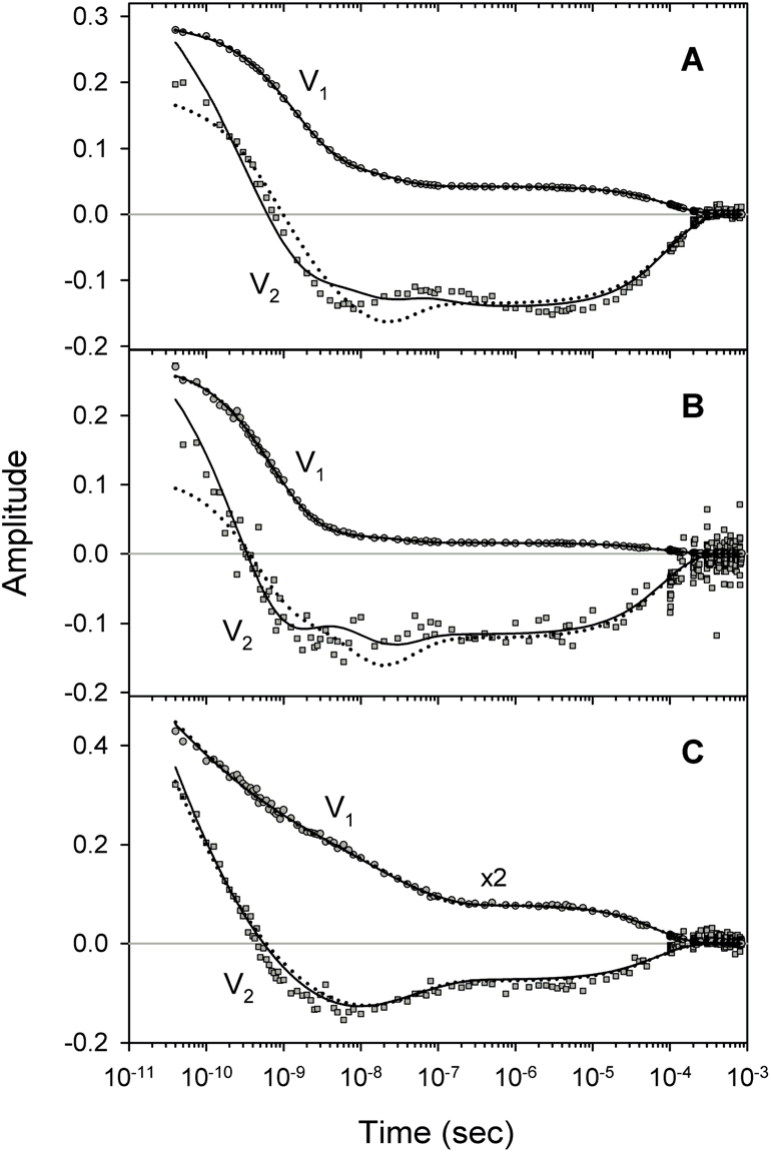
Fit to the basic (dotted lines) and extended kinetic model (solid lines). Time-dependent amplitudes, V_1_ and V_2_, shown by circles and squares, respectively, were obtained with the SVD analysis of the time-resolved spectra *D*(*λ*,*t*) measured after O_2_ photodissociation from (A) the isolated α^SH^ chains, (B) the α subunits within α_2_(Fe^2+^–O_2_)β_2_(Fe^3+^–CN) valency hybrids, and (C) the β subunits within α_2_(Fe^3+^–CN)β_2_(Fe^2+^–O_2_) valency hybrids.

A comparison of the simulated kinetic data, obtained using the resulting parameters for both models, to the experimental data, is shown in Fig. 8. Here, both the simulated and experimental data are represented as the time-dependent amplitudes V_1_ and V_2_ of the first two basis spectra, U_1_ and U_2_, obtained by the SVD analysis of the time-resolved spectra *D*(*λ*,*t*). The first amplitude vector, V_1_, is matched very well by the simulation. The second amplitude vector, V_2_, is also matched well however with some systematic deviations. The extended kinetic model shows improved goodness-of-fit as compared to the basic model (Fig. 8, solid and dotted lines, respectively). For the oxygenated α and β subunits within the Hb valency hybrids (Fig. 8B and C, respectively), the sum of squared weighted residuals, *ρ* (see ESI†), is reduced by 3% with the extended kinetic model. Whereas, for the isolated α^SH^ chains (Fig. 8A), the observed improvement of the fit is one order of magnitude higher, ρ being reduced by 50%. Such improvements of the fit support the existence of the secondary docking site(s), at least, in the α subunits.

**Fig. 9:**
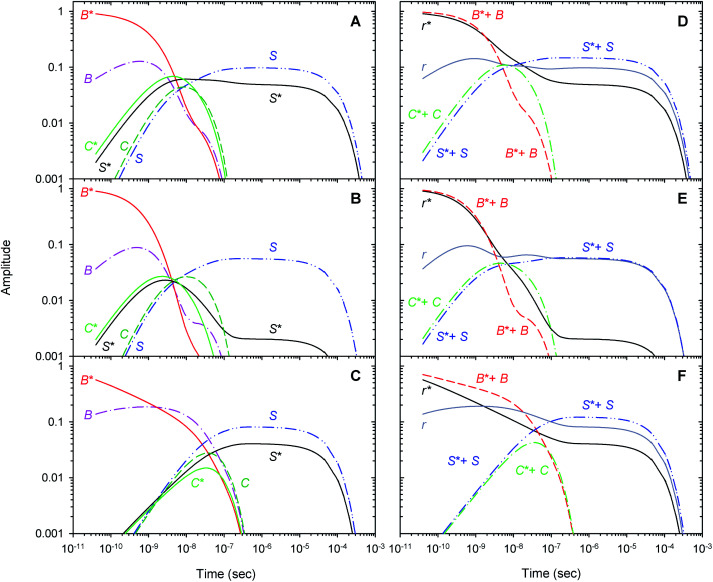
Populations of the unliganded (reagent) states (left column) and the unliganded subunits in the r* and *r* tertiary conformations (right column) predicted by the extended kinetic model for the isolated α^SH^ chains (panel (A) and (D)), for the α subunits within α_2_(Fe^2+^–O_2_)β_2_(Fe^3+^–CN) valency hybrids (panel (B) and (E)), and for the β subunits within α_2_(Fe^3+^–CN)β_2_(Fe^2+^–O_2_) valency hybrids (panel (C) and (F)).

Within the framework of the extended kinetic model, fractional populations of the unliganded (reagent) states as well as fractional populations of the unliganded subunits in each of the two different tertiary conformations, r* and *r*, were obtained and presented in Fig. 9. Additionally, the populations predicted by the extended and basic kinetic models are shown for comparison in ESI Fig. S4.† Maximum values of the fractional populations of the unliganded states C* + C and S* + S are presented in Table 5. The times at which the populations reach their maximum are presented as well. As it is seen, the maximum fractional populations of the secondary docking site(s) do not exceed 0.05 for the oxygenated α and β subunits within the valency hybrids (Table 5 [C* + C]_max_). For the isolated α^SH^ chains, the value is twice larger, being about 0.11. This means that, on the average, nearly one from every ten photodissociated O_2_ molecules visits the secondary docking site(s) in the isolated α^SH^ chains, whereas in the α and β subunits within the valency hybrids – only one from every twenty molecules. Therefore, upon incorporation of the isolated α chains into the valency hybrids, the number of photodissociated O_2_ molecules visiting the secondary docking site(s) in these subunits is decreased by a factor of ∼2. It should be noted that the maximum fractional population of the affected subunits without the captured ligand, [S* + S]_max_, is equal to the efficiency of ligand escape from the protein matrix into the environmental medium. As it is seen from Table 5, upon incorporation of the isolated α chains, the value of [S* + S]_max_ for the α subunits is also decreased by a factor of ∼2. Interestingly, the secondary docking sites in the isolated α^SH^ chains and α subunits within the valency hybrids are populated at the same time (Table 5, *t*([C* + C]_max_)). Moreover, in the valency hybrids, the maximum population of the secondary docking site(s), [C* + C]_max_, after the O_2_ photodissociation is achieved about ten times earlier in the α subunits (by ∼5 ns) than in the β subunits (by 40 ns).

Recently, to experimentally verify if the O_2_ migration occurs *via* the Xe docking sites,^18^ the O_2_ rebinding to human R-state Hb and its isolated chains has been studied under Xe pressure using a nanosecond laser flash photolysis technique.^43^ Filling internal cavities in the isolated α and β chains as well as in tetrameric R-state Hb with Xe atoms resulted in decreasing the time constant of the slowest nanosecond component of the geminate O_2_ rebinding, the time constant being several tens of nanoseconds. The observed decrease in the time constant was explained by reduction of the free internal volume accessible to O_2_ diffusion within the protein matrix after Xe insertion. Taking into account the experimental results,^43^ it can be suggested that some of the Xe binding cavities identified in the α and β subunits^18^ play a role as the secondary docking sites for photodissociated O_2_ predicted by the extended kinetic model (Scheme 2). In support of this suggestion, molecular dynamics simulations have previously demonstrated the O_2_ migration through the Xe docking sites of the isolated α chains of human Hb.^59^ Moreover, ligand diffusion tunnels in the α and β subunits of tetrameric Hb have been shown to encompass the Xe cavities regardless of the quaternary structure.^60,61^ Recently, CO sequestration in the Xe docking sites of the α and β subunits of tetrameric human Hb has been observed by X-ray crystallography using continuous irradiation by high-repetition pulsed laser light at cryogenic temperatures.^23^

### 3.7 Difference spectra between unliganded and O_2_-liganded proteins

**Fig. 10:**
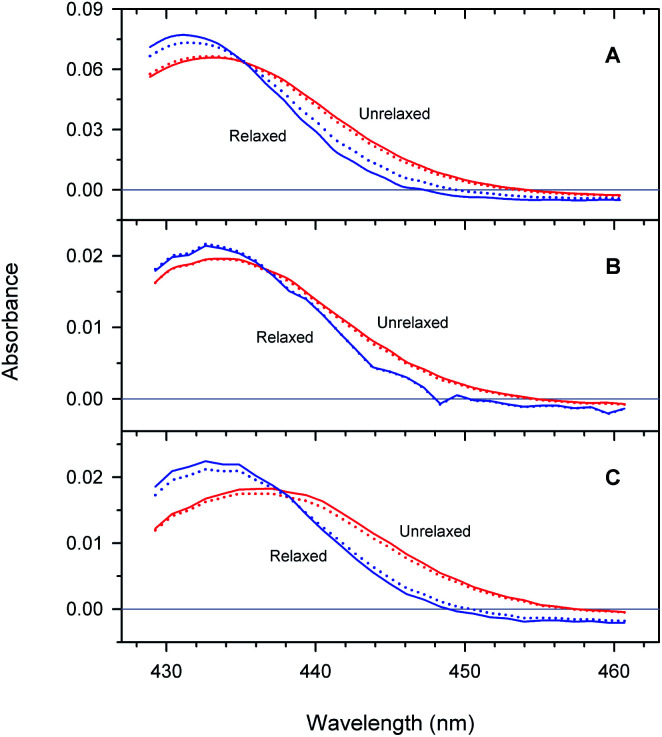
Difference spectra between unliganded and O_2_-liganded ferrous subunits in each of the two tertiary structures r* and *r* (labeled as unrelaxed and relaxed, respectively) predicted by the extended (solid lines) and basic kinetic model (dotted lines) for the isolated α^SH^ chains (A), the α subunits within α_2_(Fe^2+^–O_2_)β_2_(Fe^3+^–CN) valency hybrids (B), and the β subunits within α_2_(Fe^3+^–CN)β_2_(Fe^2+^–O_2_) valency hybrids (C).

Unliganded minus O_2_-liganded difference spectra of each of the two tertiary structures r* and *r* predicted by the extended as well as basic kinetic models are shown in Fig. 10. The maxima in the difference spectra are given in ESI Table S4.† As it is seen, in a chosen conformation, the maximum in the difference spectra depends on the type of Hb subunits as well as on their state of association. In particular, in each of the two tertiary structures, the maximum in the difference spectrum of the isolated α^SH^ chains is blue-shifted compared to that of the α subunits within the valency hybrids (Fig. 10, panel (A) and (B), respectively). Moreover, the α and β subunits within the valency hybrids are seen to be spectroscopically distinct species. In the unrelaxed r* conformation, the maximum in the difference spectrum of the α subunits is blue-shifted by about 150 cm^−1^ compared to that of the β subunits (Fig. 10, panel (B) and (C), respectively; unrelaxed). The present spectra are in an agreement with those previously obtained from the measurements of the nanosecond geminate O_2_ rebinding to the α and β subunits within Fe–Co hybrid Hbs^9^ and the bimolecular O_2_ rebinding to the α and β subunits within the native Hb in the R-state.^4^ It has been reported^4,9^ that the difference spectra of the α subunits in the Fe-containing forms are peaked at shorter wavelengths than those of the β subunits.

As it is seen from Fig. 10, the maximum in the difference spectra of all the proteins is blue-shifted upon conformational relaxation. The value of the shift depends on the type of Hb subunits as well as their state of association (ESI Table S4†). As it can be seen from Table S4,† after the O_2_ photodissociation from the α subunits within the valency hybrids, the conformational relaxation leads to the relatively small spectral changes (Δ*ν* ∼ 25 cm^−1^ within the framework of the extended kinetic model). After removing the damping imposed by the neighboring subunits (*i.e.* in the isolated α^SH^ chains), the increase in the amplitude of spectral changes is observed (Δ*ν* ∼ 100 cm^−1^). The largest shift is observed for the β subunits within the valency hybrids (Δ*ν* ∼ 175 cm^−1^).

### 3.8 Functional non-equivalence of the α and β subunits

For both kinetic models, all the parameters but one are determined rather accurately (Tables 3 and 4). The available experimental data are not sufficient to find the tertiary equilibrium constant *l* alone. For the β subunits within the valency hybrids, the parameters of the extended model do not differ significantly (no more than by a factor of 1.5) from the corresponding parameters of the basic model. Within the framework of both models, the stretched exponential parameter, β = 0.31 ± 0.02, characterizing the tertiary relaxation following the O_2_ photodissociation in the β subunits within the valency hybrids (Tables 3 and 4) is in an excellent agreement with the corresponding value, β ≈ 0.3, determined previously for the conformational relaxation upon the CO photodissociation from Hb.^12,13^ Moreover, the stretched exponential parameter of 0.41 ± 0.02 found by us for the isolated β^SH^ chains (Table 3) is very close to that of 0.45 determined for the isolated carbonmonoxy β chains of Hb.^58^ In turn, for the α subunits both in the isolated state and within the valency hybrids, the solutions obtained for the two models differ significantly in the stretched exponential parameter, *β*, in the rate constant for the geminate O_2_ rebinding to the relaxed protein, *k*_gem_(*r*), and in the rate constant for O_2_ escape from the protein, *k*_BS_. In particular, for the α subunits within the valency hybrids, the stretched exponential parameter, *β*, was found to be 0.999 ± 0.011 for the basic model, however it is 0.57 ± 0.02 for the extended model. In the former case (the basic model), when *β* is equal to unity within the experimental accuracy, an unusual exponential relaxation of tertiary structure is predicted. In the latter case (the extended model), when *β* is intermediate between zero and unity, the tertiary relaxation process is nonexponential in time. The extended model seems to produce more realistic results for the α subunits within the valency hybrids as well as for the isolated α chains. Within the framework of the extended model, the increase in the stretched exponential parameter, *β*, from 0.38 ± 0.01 to 0.57 ± 0.02 upon incorporation of the isolated α chains into the valency hybrids (Table 4) indicates more rapid equilibration of conformational substates after the O_2_ photodissociation in the α subunits within the valency hybrids as compared to the isolated α chains. The obtained results can be explained by damping the protein motions by the adjacent subunits within the valency hybrids. Moreover, the interactions between the hemoglobin subunits lead to the increase in the rate constant for the geminate O_2_ rebinding to the α subunits in the unrelaxed r* tertiary conformation (Table 4, *k*_gem_(r*)). As a result, the number of O_2_ molecules escaping from the α subunits as well as those visiting the secondary docking site(s) in these subunits is noticeably reduced (see Table 5 [S* + S]_max_ and [C* + C]_max_, respectively).

**Table 3:** Parameters obtained from the data fit to the basic kinetic model^a^

Protein	*β*	*l*	*k*(r* → *r*) (ns^−1^)	*k*_gem_(r*) (ns^−1^)	*k*_gem_(*r*) (μs^−1^)	*k*_BS_ (μs^−1^)	*k*_SB_ (ms^−1^)
αO_2_	0.85 ± 0.01	38 ± 308	0.13 ± 0.03	0.70 ± 0.01	13.3 ± 0.2	17.3 ± 0.2	23.7 ± 0.2
βO_2_	0.41 ± 0.02	2 ± 7	0.62 ± 0.75	15 ± 4	11.8 ± 0.6	8.2 ± 0.2	20.2 ± 0.3
α_2_(Fe^2+^–O_2_)β_2_(Fe^3+^–CN)	0.999 ± 0.011	29 ± 246	0.14 ± 0.04	1.37 ± 0.01	11.1 ± 0.7	13.3 ± 0.5	27.5 ± 0.6
α_2_(Fe^3+^–CN)β_2_(Fe^2+^–O_2_)	0.31 ± 0.02	4 ± 8	1.27 ± 0.55	90 ± 25	9.6 ± 0.5	8.5 ± 0.2	28.2 ± 0.5

a The uncertainties are presented as one standard error.

**Table 4:** Parameters obtained from the data fit to the extended kinetic model^a^

Protein	*β*	*l*	*k*(r* → *r*) (ns^−1^)	*k*_gem_(r*) (ns^−1^)	*k*_gem_(*r*) (μs^−1^)	*k*_BS_ (μs^−1^)	*k*_SB_ (ms^−1^)	*k*_BC_ (μs^−1^)	*k*_CB_ (μs^−1^)
αO_2_	0.38 ± 0.01	2 ± 2	0.09 ± 0.03	1.05 ± 0.02	81 ± 2	55.0 ± 0.4	17.3 ± 0.1	78 ± 1	59 ± 1
α_2_(Fe^2+^–O_2_)β_2_(Fe^3+^–CN)	0.57 ± 0.02	27 ± 164	0.115 ± 0.037	1.68 ± 0.03	96 ± 8	43.8 ± 0.9	18.1 ± 0.4	55 ± 2	41 ± 2
α_2_(Fe^3+^–CN)β_2_(Fe^2+^–O_2_)	0.31 ± 0.01	4 ± 7	1.8 ± 0.8	120 ± 42	14.4 ± 0.9	10.8 ± 0.4	26.0 ± 0.4	8 ± 2	16.6 ± 3.4

a The uncertainties are presented as one standard error.

**Table 5:** Maximal populations of the unliganded states C* + C and S* + S as well as the times at which the maximal populations are achieved

Protein	[C* + C]_max_ (× 10^−2^)	*t*([C* + C]_max_) (ns)	[S* + S]_max_^a^ (× 10^−2^)	*t*([S* + S]_max_)^a^ (ns)
αO_2_	10.9	6	14.7 (14.6)	200 (250)
α_2_(Fe^2+^–O_2_)β_2_(Fe^3+^–CN)	4.6	4.5	5.8 (5.8)	250 (300)
α_2_(Fe^3+^–CN)β_2_(Fe^2+^–O_2_)	4.2	40	12.1 (12.7)	500 (400)

a The data obtained by the extended model are given without parenthesis, while those obtained by the basic model are presented in parenthesis.

For all the studied proteins, the tertiary transition rate, *k*(r* → *r*), the rate constant for the geminate O_2_ rebinding to the unrelaxed protein, *k*_gem_(r*), and the rate constant for O_2_ escape from protein, *k*_BS_, produced by the extended model, do not differ significantly from the corresponding parameters of the basic model. Within the framework of both models, functional non-equivalence of the α and β subunits within the valency hybrids is predicted. Since the extended model provides more realistic results, we will hereafter discuss only the data, obtained *via* this model. Namely, for the β subunits within the valency hybrids, the rate constant for the geminate O_2_ rebinding to the unrelaxed structure, *k*_gem_(r*), and the tertiary transition rate, *k*(r* → *r*), are greater by a factor of 70 ± 25 and 16 ± 9, respectively, than the corresponding values for the α subunits within the hybrids (Table 4). For the β subunits, the larger rate constant *k*_gem_(r*), describing the O_2_ rebinding from within the primary docking site, can be explained, at least in part, by a closer location of the primary docking site to its binding site in these subunits compared to that in the α subunits.^15^ Recently, time-resolved Laue crystallography of photolyzed carbonmonoxy Hb in the R-state has revealed the populations of CO in the binding and primary docking sites in the heme pockets of both the α and β subunits, the primary docking site in the β subunits being at about 0.25 Å closer to its binding site.^15^ The obtained correlation between the rate constant of the O_2_ rebinding from within the primary docking site *k*_gem_(r*) and the ligand displacement from the heme binding site^15^ suggests the distal control of the geminate O_2_ rebinding.

In the present experiment, the tertiary conformational relaxation (r* → *r*) following the O_2_ photodissociation from the ferrous heme iron in each Hb subunit leads to a significant slowing down of the geminate O_2_ rebinding from within the primary docking site. For the β subunits within the valency hybrids, the rate constant for the geminate O_2_ rebinding, *k*_gem_, slows by a factor of 8300 ± 3000 (Table 4). In turn, for the α subunits within the valency hybrids and the isolated α^SH^ chains, the conformational relaxation slows the geminate O_2_ rebinding by a factor of 18 ± 2 and 13 ± 1, respectively (Table 4). Therefore, upon the relaxation of the tertiary structure, the decrease in the rate constant for the geminate O_2_ rebinding from within the primary docking site of the β subunits within the valency hybrids is more than one order in magnitude larger than the corresponding changes obtained for the α subunits. The obtained data reveal significant α/β differences in both the geminate O_2_ rebinding and concomitant structural changes. For the individual α and β subunits within Hb in the R-state like conformation, the non-equivalent decrease in the rate constant of the O_2_ rebinding from within the primary docking site is observed at the conformational relaxation following the O_2_ photodissociation.

## 4 Conclusions

Our results demonstrate that the oxy-cyanomet valency hybrids, α_2_(Fe^2+^–O_2_)β_2_(Fe^3+^–CN) and α_2_(Fe^3+^–CN)β_2_(Fe^2+^–O_2_), are good models for the α and β subunits within native R-state oxyhemoglobin with respect to the O_2_ rebinding and conformational relaxation following the O_2_ photodissociation. For the R-state oxyhemoglobin, the O_2_ rebinding properties and spectral changes following the O_2_ photodissociation can be adequately described as the sum of those for the α and β subunits within the valency hybrids. The magnitude of the spectral shift, Δ*ν*, of the positive peak near 434 nm as well as the position of this peak during the conformational relaxation following the O_2_ photodissociation in the R-state oxyhemoglobin are similar to those obtained by averaging the corresponding values for the two valency hybrids. The values of the efficiency of O_2_ escape from the α and β subunits within the valency hybrids were found to be in excellent agreement with the ones obtained for the corresponding α and β subunits within native oxyhemoglobin in the R-state.^22^ The isolated β^SH^ chains (hemoglobin H) show similar behavior to the β subunits within the valency hybrids and can be used as a model for the β subunits within the R-state oxyhemoglobin. At the same time, the isolated α^SH^ chains behave differently to the α subunits within the valency hybrids.

Significant functional non-equivalence of the α and β subunits within the Hb valency hybrids in both the geminate O_2_ rebinding and concomitant structural relaxation was revealed. In particular, the α subunits within α_2_(Fe^2+^–O_2_)β_2_(Fe^3+^–CN) show one dominant rapid and one slower scarcely observed geminate phases. While the β subunits within α_2_(Fe^3+^–CN)β_2_(Fe^2+^–O_2_) show two distinct geminate phases with the similar fractional contributions. It is the β subunits within the tetrameric valency hybrids that have the fastest geminate phase. To describe the geminate O_2_ rebinding in the ferrous Hb subunits as well as the nonexponential tertiary relaxation within the R quaternary structure, we followed the basic kinetic model,^12^ which contains the minimal set of required states. Moreover, to account for the O_2_ migration between the primary and secondary docking site(s), the basic kinetic model was extended. Within the framework of both models, functional differences of the α and β subunits within the valency hybrids is observed. Namely, for the β subunits, the rate constant for the geminate O_2_ rebinding to the unrelaxed structure, *k*_gem_(r*), and the tertiary transition rate, *k*(r* → *r*), were found to be greater than the corresponding values for the α subunits. For the β subunits, the larger rate constant *k*_gem_(r*) is explained, at least in part, by the closer location of the primary docking site to its binding site in these subunits compared to that in the α subunits, suggesting the distal control of the geminate O_2_ rebinding. The tertiary relaxation following the O_2_ photodissociation in the α and β subunits was found to decrease the rate constant for the geminate O_2_ rebinding, this effect being more than one order of magnitude greater for the β subunits than for the α subunits. Moreover, the α and β subunits within the valency hybrids were revealed to be spectroscopically distinct species. The maximum in the difference spectrum of each Hb subunit is blue-shifted upon the conformational relaxation. The largest shift of the maximum is observed for the β subunits. The temporal evolution of the spectral changes, observed in the present experiment for both the ferrous α and β subunits, is rather well correlated with the published data for conformational relaxation involving the iron-proximal histidine bond following the ligand photodissociation. The obtained correlation provided evidence for the modulation of the O_2_ rebinding to the individual α and β subunits within human Hb in the R-state structure by the intrinsic heme reactivity through a change in proximal constraints upon the relaxation of the tertiary structure on a picosecond to microsecond time scale.

## Author contributions

SVL, IVS and BMD initiated the investigations. SVL, MVP and SNG prepared the protein samples. SVL and IVS performed the transient absorption experiments. SVL analyzed the data, performed modelling and wrote the manuscript. All authors contributed to discussing the results and reviewing the manuscript.

## Conflicts of interest

There are no conflicts to declare.

## Supplementary Material

SC-012-D1SC00712B-s001

SC-012-D1SC00712B-s002

SC-012-D1SC00712B-s003

SC-012-D1SC00712B-s004

SC-012-D1SC00712B-s005

SC-012-D1SC00712B-s006
