# Supplementary figures and images for: Towards understanding non-equivalence of α and β subunits within human hemoglobin in conformational relaxation and molecular oxygen rebinding
Source: Chem Sci. 2021 Apr 15;12(20):7033–47. doi: 10.1039/d1sc00712b (PMC8153241; doi:10.1039/d1sc00712b)

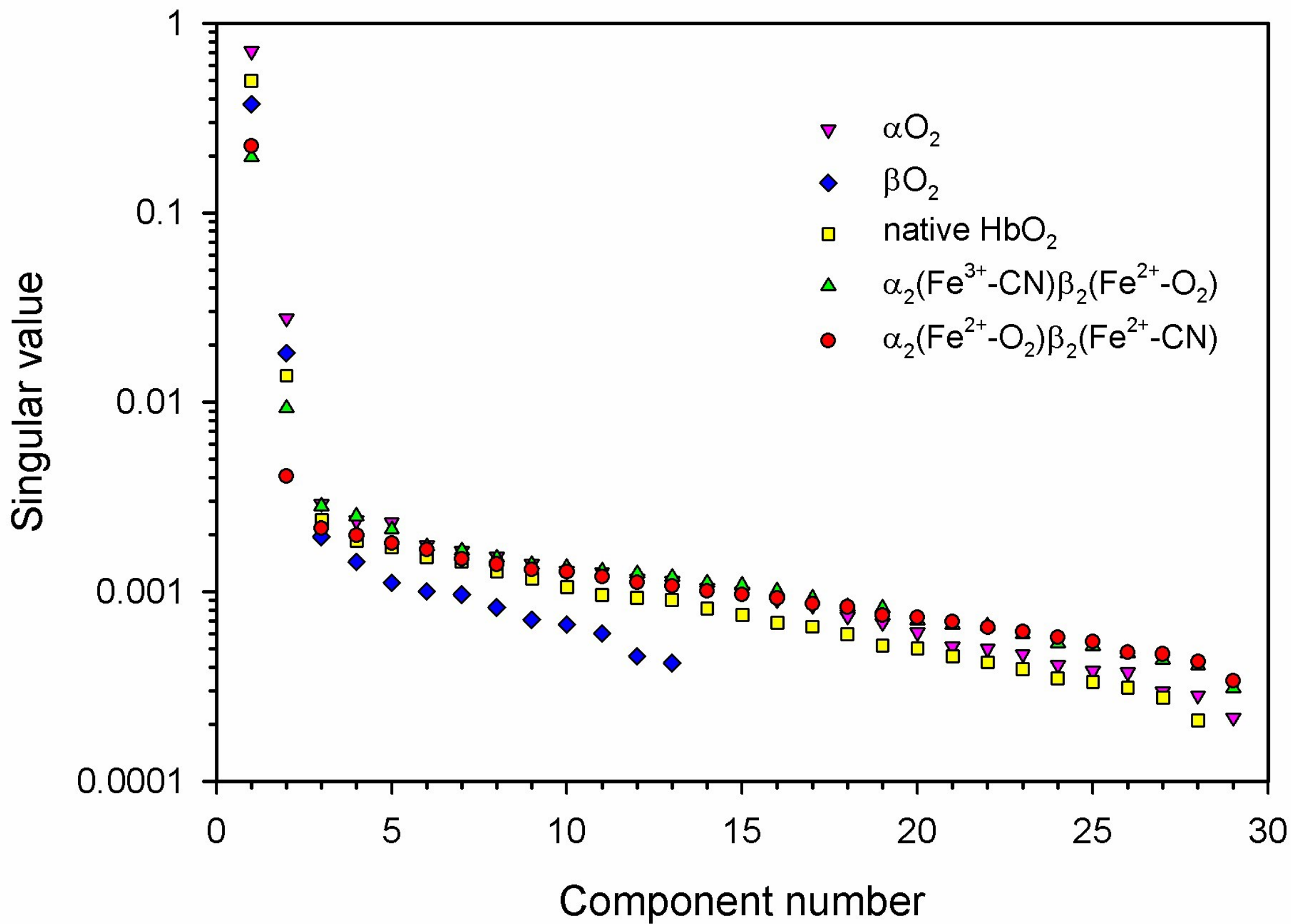

Supplement: SC-012-D1SC00712B-s002 [file SC-012-D1SC00712B-s002.pdf]

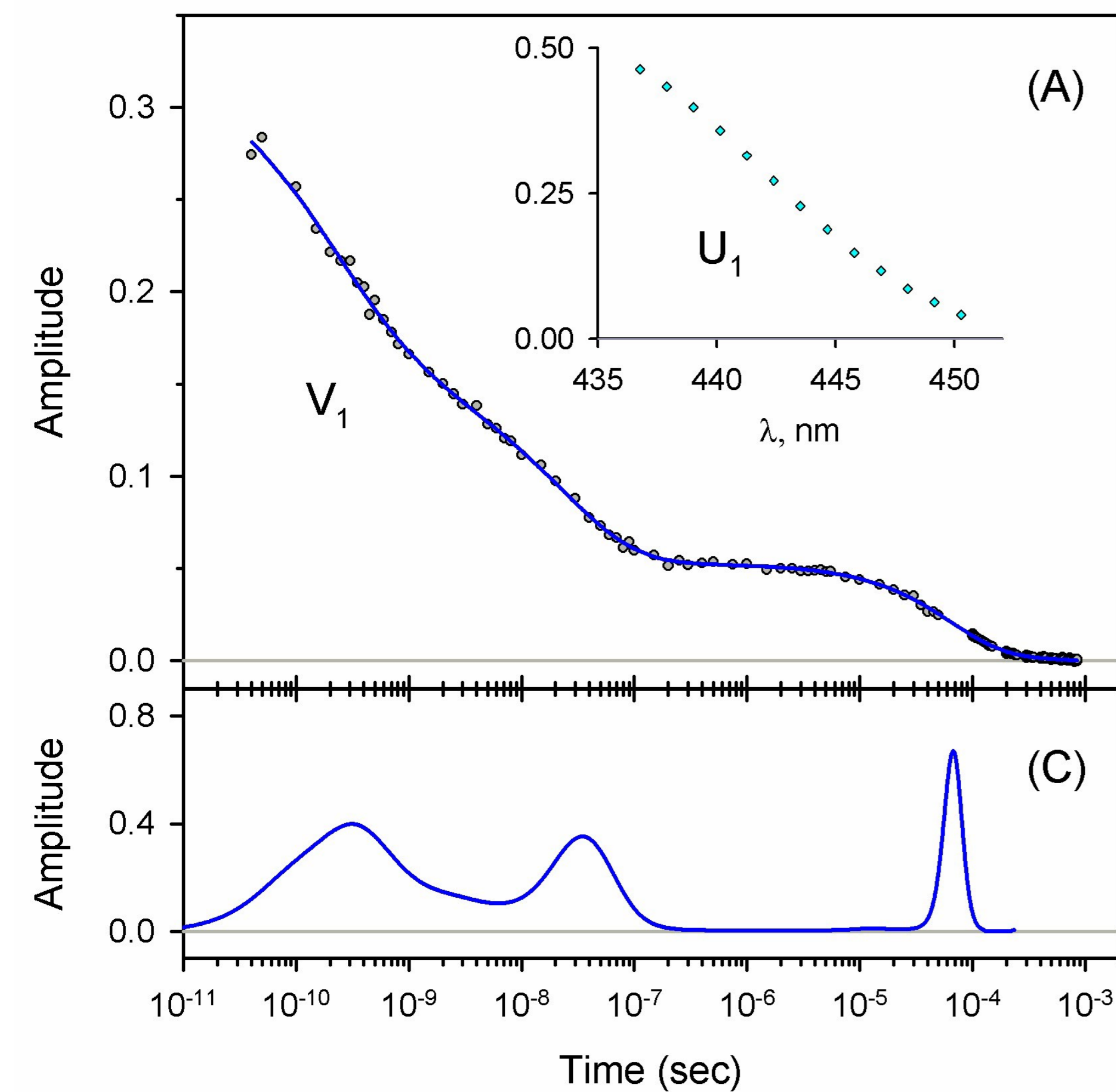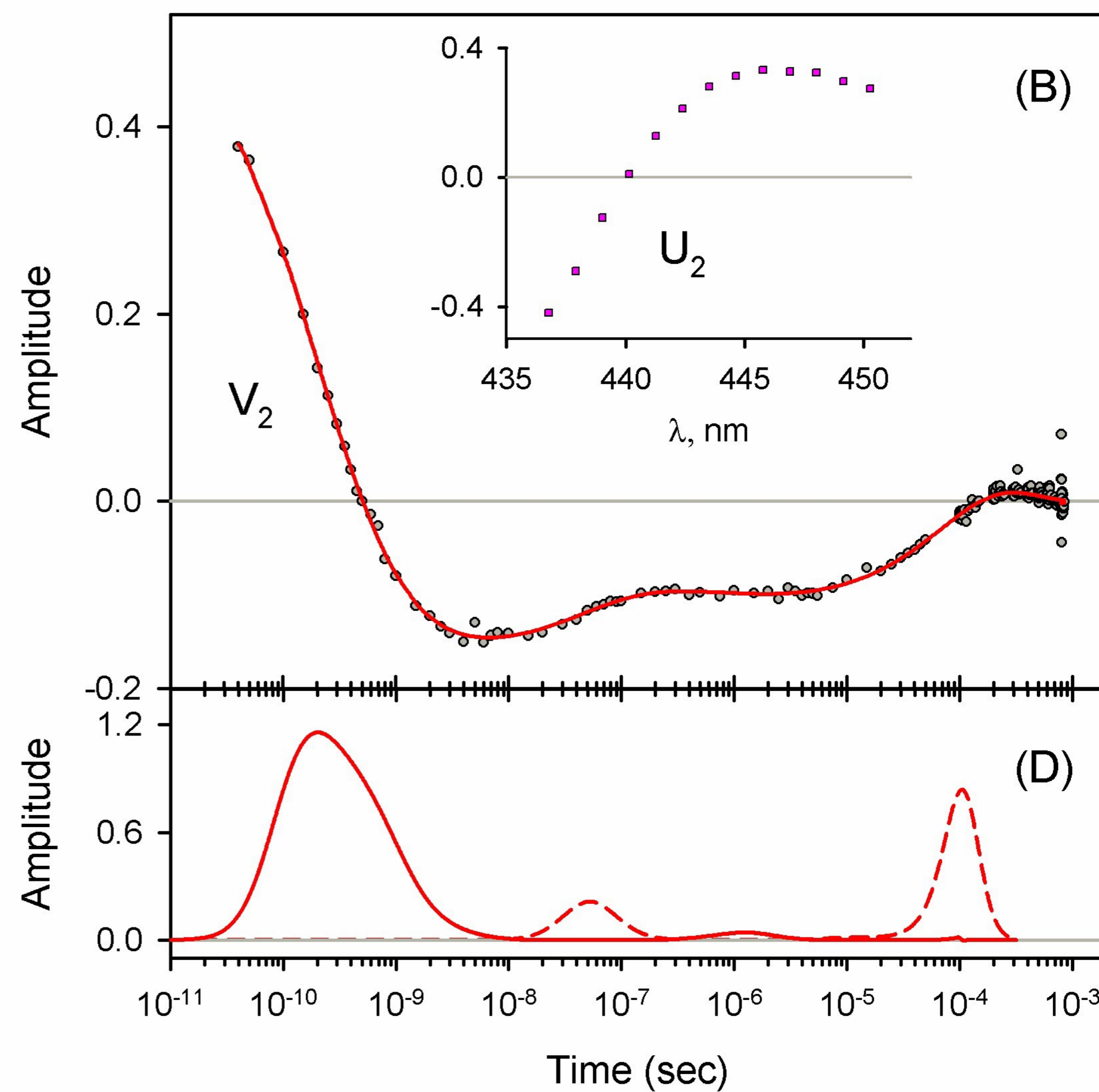

Supplement: SC-012-D1SC00712B-s003 [file SC-012-D1SC00712B-s003.pdf]

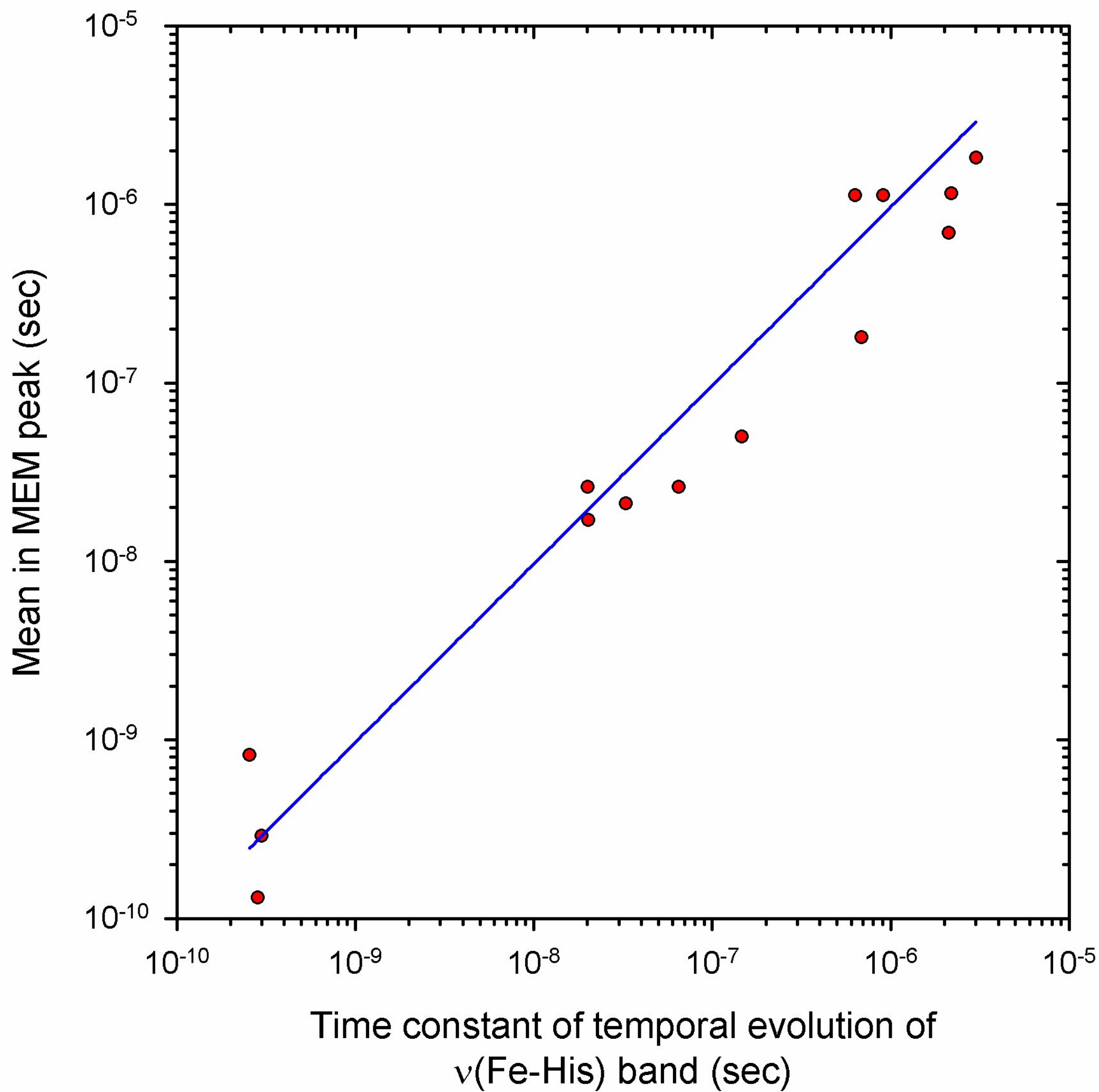

Supplement: SC-012-D1SC00712B-s004 [file SC-012-D1SC00712B-s004.pdf]

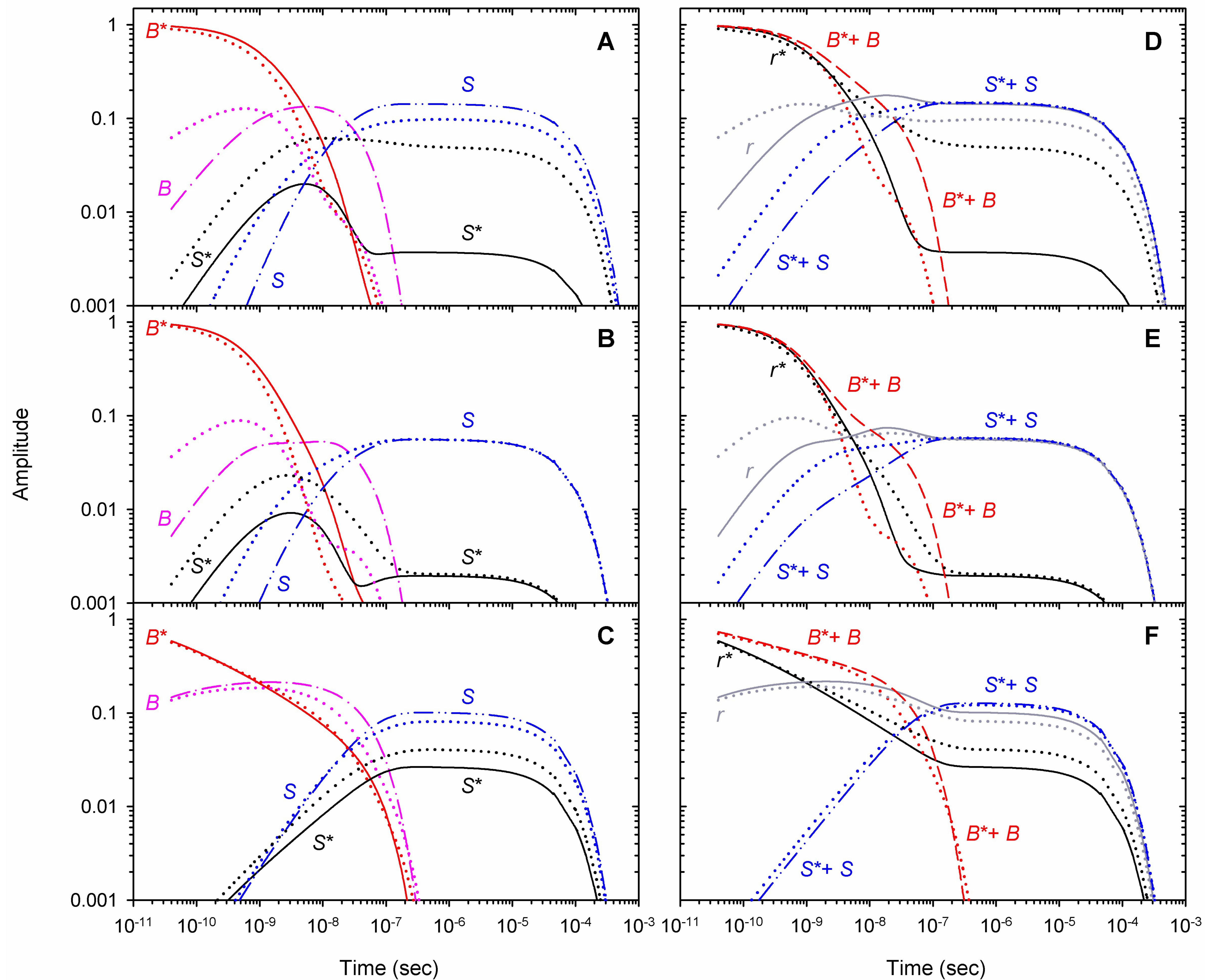

Supplement: SC-012-D1SC00712B-s005 [file SC-012-D1SC00712B-s005.pdf]
