# Supplementary material for: Towards understanding non-equivalence of α and β subunits within human hemoglobin in conformational relaxation and molecular oxygen rebinding
Source: Chem Sci. 2021 Apr 15;12(20):7033–47. doi: 10.1039/d1sc00712b (PMC8153241; doi:10.1039/d1sc00712b)
Supplement: SC-012-D1SC00712B-s006 [file SC-012-D1SC00712B-s006.pdf]

Electronic Supplementary Material (ESI) for Chemical Science.
This journal is © The Royal Society of Chemistry 2021

Supplementary Information for

Towards understanding non-equivalence of α and β subunits within human hemoglobin in conformational relaxation and molecular oxygen rebinding

Sergei V. Lepeshkevich,[*a] Igor V. Sazanovich,[*b] Marina V. Parkhats,[a] Syargey N. Gilevich[c] and Boris M. Dzhagarov[a]

[a]B.I. Stepanov Institute of Physics, National Academy of Sciences of Belarus, 68 Nezavisimosti Ave, Minsk 220072, Belarus. E-mail: s.lepeshkevich@ifanbel.bas-net.by
[b]Central Laser Facility, Research Complex at Harwell, STFC Rutherford Appleton Laboratory, Harwell Campus,OX11 0QX, UK. E-mail: igor.sazanovich@stfc.ac.uk
[c]Institute of Bioorganic Chemistry, National Academy of Sciences of Belarus, 5 Academician V.F. Kuprevich Street, Minsk 220141, Belarus

Table of Contents

Supplementary Methods S3

1. Preparation of isolated hemoglobin chains S3
2. Preparation of hemoglobin valency hybrids S3
3. Time-resolved spectroscopy S4
4. Singular value decomposition (SVD) analysis S5
5. Maximum entropy method (MEM) analysis S5
6. Simulation of experimental data S6

Supplementary Figures S8

Supplementary Tables S11

Supplemental References S14

Supplementary Methods

1 Preparation of isolated hemoglobin chains

Human Hb was purified from freshly drawn blood. The isolated α and β chains were subsequently obtained by the *p*-mercuribenzoate (PMB) method[1] with minor modifications. The isolated chains with bound PMB (α^{PMB} and β^{PMB} chains) were obtained in either the oxy or the carbonmonoxy form. Separation of the PMB-reacted chains was carried out on a column with DEAE-Sepharose CL-6B (GE Healthcare) equilibrated with 20 mM Tris HCl buffer, pH 8.1. Elution was carried out by a linear gradient produced by mixing equal volumes of 20 and 380 mM Tris HCl buffers, both at pH 8.1, in a gradient mixer GM-1 (GE Healthcare). The first eluted fraction contained the α^{PMB} chains, while the other main fraction, eluted later, contained the β^{PMB} chains. The isolated α and β chains were regenerated to their -SH forms (α^{SH} and β^{SH} chains) using dithiothreitol (DTT).[2] PMB was removed from the α chains after incubation with DTT for a few minutes and subsequent passage through a Sephadex G-25 column equilibrated with 5 mM Tris HCl buffer, pH 8.1. The β^{PMB} solution was incubated with DTT for 30 minutes on ice and, subsequently, applied to a DEAE-Sepharose CL-6B column equilibrated with 10 mM Tris HCl buffer, pH 8.1. The β^{PMB} chains stick at the top of the column. The column was washed with the same buffer containing 4 mM DTT for ~2.5 hrs and left overnight. The next morning, the column was washed with 140 mM Tris HCl buffer, pH 8.1, for ~2 hrs, then the mercury-free β^{SH} chains were eluted with 220 mM Tris HCl buffer at the same pH. All the procedures were carried out at 4°C. All the buffers used for the sulfhydryl groups regeneration were purged carefully with nitrogen. The molecular weights of the α and β chains were verified by electrospray ionization mass spectroscopy.

2 Preparation of hemoglobin valency hybrids

The Hb valency hybrids of the type $\alpha_2(Fe^{2+}\text{-}O_2)\beta_2(Fe^{3+}\text{-}CN)$ and $\alpha_2(Fe^{3+}\text{-}CN)\beta_2(Fe^{2+}\text{-}O_2)$ were prepared by mixing the isolated chains with free -SH groups in the oxy form with their partner chains in the cyanomet form.[3] The cyanomet forms of the isolated α^{SH} and β^{SH} chains were obtained from the carbonmonoxy-derivatives by oxidation with potassium ferricyanide in the presence of sodium cyanide. The complete oxidation of the isolated chains with the concomitant formation of the cyanomet-derivatives was controlled spectrophotometrically. Ferri- and ferrocyanide were removed by gel filtration on a Sephadex G-25 column equilibrated with 50 mM Tris HCl buffer, pH 8.2. All the procedures were carried out at 4°C. The valency hybrids were prepared within one day before experimental measurements. To ensure as complete recombination as possible of the oxygenated partner, an excess of the cyanomet chains (between

20 and 50%) was added. The valency hybrids were not separated from the unrecombined cyanomet chains, which were spectroscopically inactive in the present O_2 rebinding studies in a specially chosen time frame (see Section 3.1 in the main text).

3 Time-resolved spectroscopy

Picosecond to millisecond time-resolved spectra in the visible (Soret) region were measured on the ULTRA[4] apparatus in the Time-Resolved Multiple Probe Spectroscopy mode[5] at the Central Laser Facility (STFC Rutherford Appleton Laboratories, Harwell, UK). The time-resolved multiple-probe spectrometer comprises two Ti:sapphire amplifiers with different repetition rates (1 and 10 kHz, respectively). The 1 kHz Ti:sapphire amplifier (Spitfire XP, Spectra-Physics, USA) was used to pump Optical Parametric Amplifier (TOPAS, Light Conversion, Lithuania) to provide 1 kHz, 100 fs, 543 nm pump pulses. These pulses were attenuated to deliver 1 μJ at the sample. The 10 kHz Ti:sapphire amplifier (Thales Laser) was applied as a probe source, producing 0.8 mJ output with 40 fs pulse duration, at 800 nm. Part of the 10 kHz laser output beam was used to generate a white-light continuum in a 2 mm CaF_2 plate, being continuously rastered to avoid colour centre formation and to improve pulse-to-pulse stability in the probe. The white-light continuum was passed through the photoexcited region of the sample and dispersed with the grating spectrograph (0.25 m *f*/4 DK240, Spectral Products) and detected using a linear silicon array (Quantum Detectors, UK). In front of the spectrograph, an appropriate notch filter was placed in order to remove scatter from the excitation beam. The transient absorption spectra were calibrated using WCT-2065 calibration standard.

Both the 1 and 10 kHz amplifiers were optically synchronized by sharing the same seed from a single Ti:sapphire 20 fs, 68 MHz oscillator laser (Synergy, Femtolaser, Germany). The seed beam was delayed with a tuneable optical delay line before the 1 kHz amplifier to cover the time delay range of 100 fs – 14.7 ns between pump and probe pulses. To go beyond 14.7 ns up to 100 μs, subsequent oscillator seed pulses were selected accompanied by the appropriate setting of the optical delay line. The probe pulses, continuously arriving to the sample every 100 μs, cover the time range of 1 ms between subsequent excitation pulses.

The pump and probe beams were focused to ~150 and 80 μm diameter spot size at the sample, respectively. The relative polarizations of the pump and probe beams were adjusted to 54.7° for "magic angle" measurements. Samples were raster scanned in the *x* and *y* directions to minimize photodamage and re-excitation effects. The raster scan settings used were ca. 5 mm raster span and rastering frequency was about 2-3 Hz. With such rastering speed, it takes between 5 to 10 ms to move the sample across the size of the excitation spot. As the slowest processes detected in the experiment had lifetimes of the order of few hundred microsecond,

raster scanning did not noticeably affect the recorded kinetics. The sample solutions were contained in quartz cells of 1 mm optical pathlength. During the measurements, the time delays were scanned in the random order to avoid systematic effects in the data due to possible sample degradation. At each time delay, the present spectra were collected for 3 seconds exposure time with both the pump and probe beams present at the sample. The data acquisition was repeated 5 times to average the data. The time-resolved spectrometer[4] used here allows to detect transient absorption spectra in the Soret region (from about 430 to 460 nm) in the time range from 1 ps up to 800 μs after the laser photoexcitation with a sensitivity of 10^{-5} absorbance units.

4 Singular value decomposition (SVD) analysis

The measured transient absorption spectra at different time delays, $D(\lambda,t)$, can be viewed as the columns of an $m \times n$ matrix **D**, where m is the number of spectral points λ, n is the number of time delays t. The time-resolved difference spectra for each studied heme protein were subjected to singular value decomposition (SVD) which transforms the $m \times n$ data matrix **D** into a product of three matrices, $\mathbf{D} = \mathbf{U}\,\mathbf{S}\,\mathbf{V}^{\mathrm{T}}$, where **U** is an $m \times n$ matrix of m linearly independent, orthonormal basis spectra U_r ($r = \overline{1,m}$), **S** is an $n \times n$ diagonal matrix with non-negative elements called singular values, $\mathbf{V}^{\mathrm{T}}$ is the transpose of an $n \times n$ matrix **V** having n orthonormal kinetic vectors V_c ($c = \overline{1,n}$), *i.e.* the time-dependent amplitudes of the basis spectra.[6] In the present application, only few basis spectra are expected to make significant contribution to **D**. The remainder can be considered as noise. To determine the contribution of the basis spectra, the magnitudes of their singular values and the autocorrelation of the time-dependent amplitudes of the corresponding basis spectra were analyzed.

5 Maximum entropy method (MEM) analysis

The obtained time-dependent amplitudes of the basis spectra were subjected to the maximum entropy method (MEM) analysis which extracts model independent lifetime distributions from the kinetics.[7] To analyze the time-dependent amplitudes in terms of distributed lifetimes, the program MemExp (version 3.0)[8,9] was used. One or two distributions of effective log-lifetimes, $g(\log\tau)$ and $h(\log\tau)$, were extracted from the data. The fit F_i to datum D_i at time t_i can be written as

$$F_i = D_0 \int_{-\infty}^{+\infty} (g(\log\tau) - h(\log\tau)) e^{-t_i/\tau} d\log\tau \qquad \text{(S1)}$$

where D_0 is a normalization constant, $g(\log\tau)$ and $h(\log\tau)$ are the lifetime distributions that correspond to decaying and rising kinetics, respectively. The quality of the fit was evaluated by the χ^2 value,[10] the correlation length of the residuals, τ_c,[8] as well as the values of Ratio[11] and

TEST.[12] To improve the fidelity of the recovered distributions as well as to determine which features in the lifetime distributions are required by the data, the fit was performed with the MEM using different prior distributions.[8,9] Namely, uniform distributions as well as distributions derived by uniform or differential blurring of intermediate MEM results were used as the prior.[8,9] Moreover, fits to the data were performed several times using different expected lifetime limits at a maximum number of anticipated lifetimes.

6 Simulation of experimental data

In the present work we performed modeling the data matrix $\tilde{\mathbf{D}}$ so that to produce a set of time-dependent species populations (which comprise a matrix **P**) and a set of corresponding species spectra (which comprise a matrix **S**), such that the product **SP** provides the best approximation to the matrix $\tilde{\mathbf{D}}$, i.e. $\tilde{\mathbf{D}} \approx \mathbf{SP}$. **P** is a $2 \times n$ matrix whose rows contain the total populations of unliganded subunits in each of the two tertiary structures r^* and r. **S** is a $m \times 2$ matrix whose columns contain difference spectra between unliganded and O_2-liganded ferrous subunits in each of the two tertiary structures r^* and r. Each difference spectrum is assumed to be a linear combination of the first two basis spectra, U_1 and U_2. Moreover, to quantitatively relate the experimental data to the kinetic models (see Section 3.5 in the main text), the elements of the first amplitude vector, V_1, obtained from the SVD analysis (see Section 3.2 in the main text), are considered to be proportional to the time-dependent total population of the unliganded subunits in all the tertiary structures. The procedure for simulating the data matrix $\tilde{\mathbf{D}}$ first specifies a set of parameters for the chosen model, then solves the differential equations for the specified set of model parameters over a required set of times using the appropriate initial conditions to produce the species population matrix **P**, and finally creates the matrix **S**, containing the corresponding species spectrum.

The fitting procedure adjusts the parameters for the chosen model to minimize the sum of squared weighted residuals, ρ. The sum ρ consists of two parts obtained from comparison of simulated outputs with two experimental data sets: (1) the time-resolved spectra and (2) the total population of unrecombined deoxyhemes. The first part is the sum of squares of all the elements of the residual matrix $\tilde{\mathbf{D}} - \mathbf{SP}$. The second one is the sum of squares of all the elements of a residual array obtained by subtracting the first amplitude vector, V_1, from an array containing a properly normalized sum of all time-dependent deoxyheme state populations provided by the solutions of the differential equations for the chosen kinetic model.

A sequential Monte Carlo simulation annealing algorithm[13] was used to find a local minimum for the sum of squared weighted residuals, ρ, in parameter space. The Metropolis criterion[14] for acceptance was employed in which ρ was treated as the “energy”. Monte Carlo

steps resulting in ρ decrease ($\rho_{new} < \rho_{previous}$) were always accepted, while steps involving in ρ increase ($\rho_{new} > \rho_{previous}$) were accepted with a probability $\exp(-(\rho_{new} - \rho_{previous})/\rho_T)$, where ρ_T is the simulation "temperature" chosen to be dependent on the number of Monte Carlo steps, k, as follows: $\rho_T = n(m + 1)(1000/k)^{1/2}$. Here, $n(m + 1)$ equals to the total number of elements both in the $m \times n$ data matrix $\tilde{\mathbf{D}}$ and in the $1 \times n$ first amplitude vector, V_1. For every kinetic model describing conformational changes and O_2 rebinding in every studied heme protein, a dozen distinct simulations using different sets of initial parameters were performed, each simulation involving up to 55 thousand Monte Carlo steps among which 5 through 25 thousand steps were successful. Each set of parameters that minimized the residuals by this procedure was then used as starting values for minimization using a Marquardt-Levenberg algorithm.[15] Finally, each such procedure yielded an approximate minimum for the sum of squared weighted residuals, ρ, in parameter space. The model parameters obtained in the distinct simulations with the smallest values of ρ are considered to be the solution.

Supplementary Figures

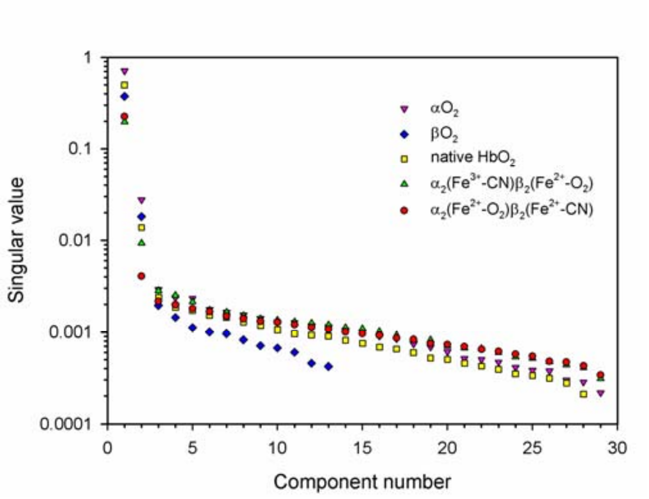

Fig. S1 Singular values from the diagonal matrix **S** as determined by SVD of the data matrix **D**. All singular values, $s_{i,i}$ ($i = \overline{1,n}$), obtained for every studied protein, are presented in Figure by plotting singular values, $s_{i,i}$, in log scale versus component number, i. As it is seen, first two components stand out above the others, which, in their turn, form almost a straight line. The rank of matrix **D** is estimated as two by identification of two singular value components above the systematic decline.

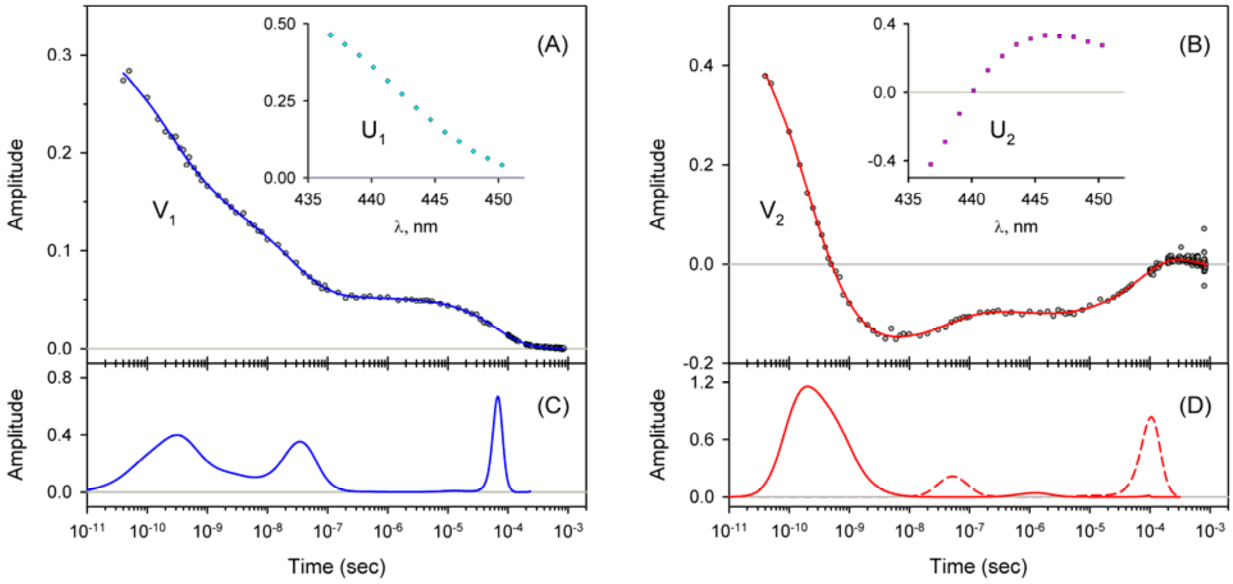

Fig. S2 Singular value decomposition of the time-resolved spectra $D(\lambda,t)$ measured after O_2 photodissociation from the isolated β^{SH} chains. Description of each panel and symbols used is the same as for Fig. 4.

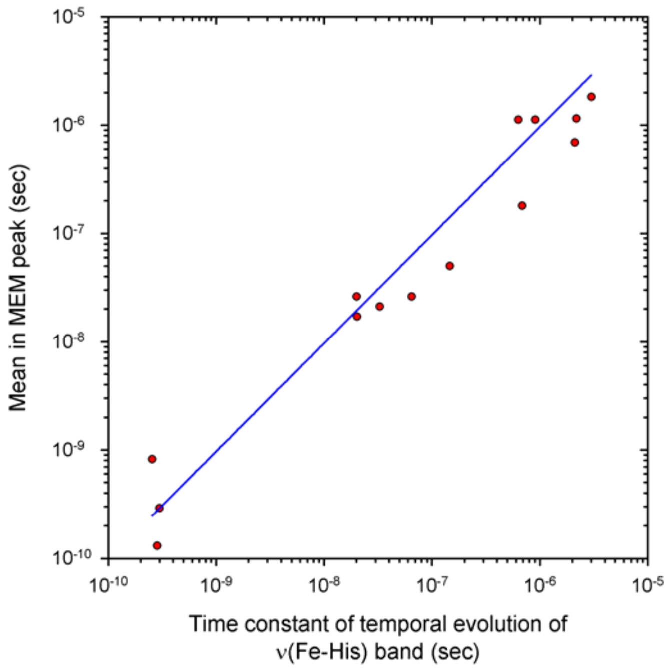

Fig. S3 Correlation between time constants of temporal changes in the ν(Fe-His) frequency following the CO photodissociation and the means of the lifetime distribution peaks extracted from the second amplitude vector, V_2. The time constants of temporal evolution of the ν(Fe-His) band were taken from literature[16–20] and are listed in Table S2 together with the corresponding data characterizing the lifetime distributions extracted from the second amplitude vector, V_2. Solid line represents fit to linear function of $y = \alpha x$ with the result of $\alpha = 0.97 \pm 0.06$.

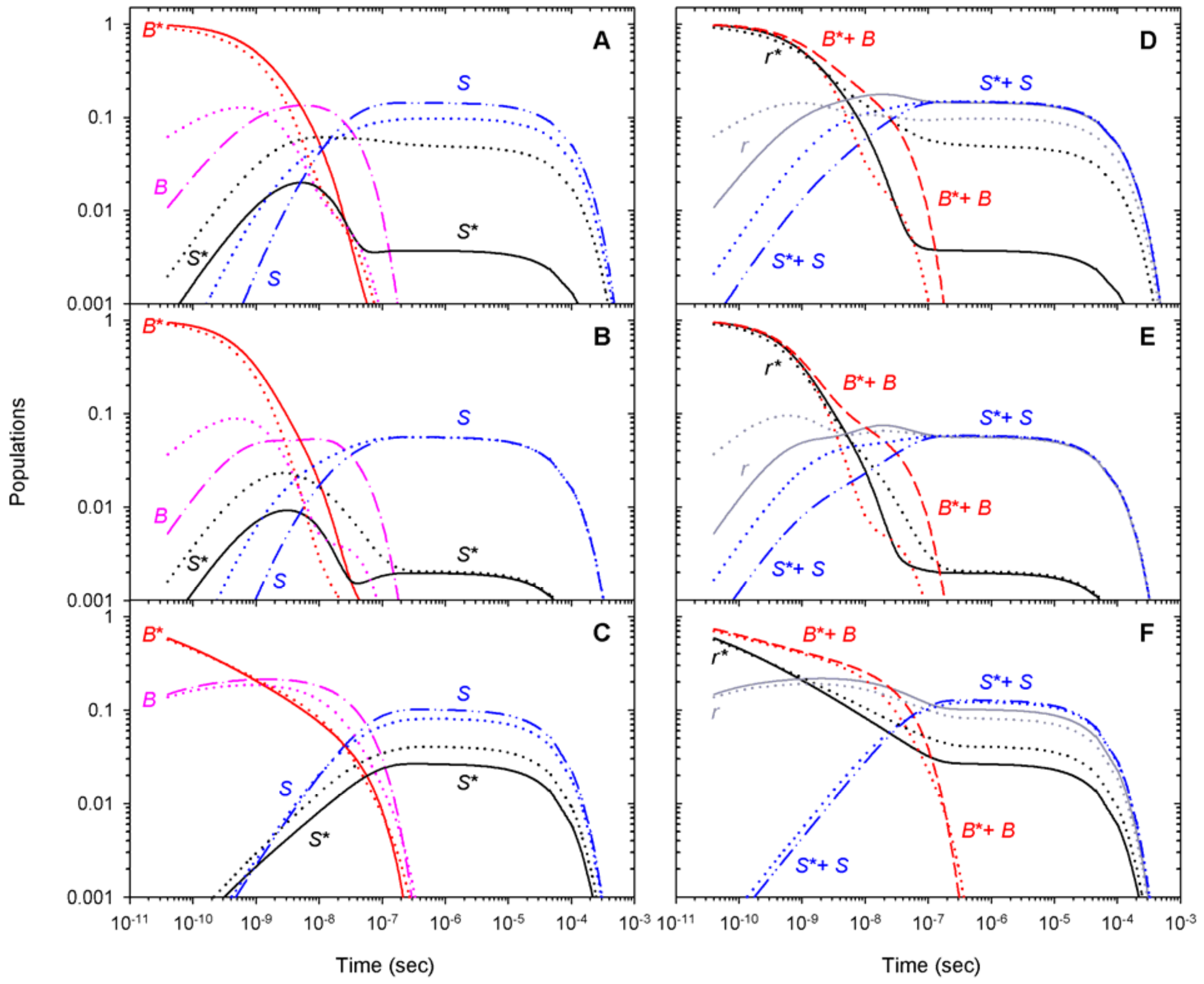

Fig. S4 Populations of the unliganded (reagent) states (left column) and the unliganded subunits in the r^* and r tertiary conformations (right column) predicted by the basic kinetic model for the isolated α^{SH} chains (panel (A) and (D)), for the α subunits within $\alpha_2(Fe^{2+}\text{-}O_2)\beta_2(Fe^{3+}\text{-}CN)$ valency hybrids (panel (B) and (E)), and for the β subunits within $\alpha_2(Fe^{3+}\text{-}CN)\beta_2(Fe^{2+}\text{-}O_2)$ valency hybrids (panel (C) and (F)). The populations obtained within the basic kinetic model are shown by lines. Corresponding populations predicted by the extended kinetic model are shown by dotted lines of respective colors for comparison.

Supplementary Tables

Table S1 Magnitudes of the spectral shift of the positive peak near 434 nm in the transient spectra following O_2 photodissociation from the heme proteins

Protein	$\nu(0)^a$ (cm^{-1})	$\nu(\infty)^b$ (cm^{-1})	$\Delta\nu$ (cm^{-1})
αO_2	23104.5 ± 1.3	23172 ± 7	68 ± 7
βO_2	22990 ± 5	23059 ± 8	69 ± 9
native HbO_2	23031 ± 5	23078 ± 6	47 ± 8
$\alpha_2(Fe^{2+}\text{-}O_2)\beta_2(Fe^{3+}\text{-}CN)$	23063 ± 3	23100 ± 14	37 ± 14
$\alpha_2(Fe^{3+}\text{-}CN)\beta_2(Fe^{2+}\text{-}O_2)$	22981 ± 3	23051 ± 9	70 ± 9

The peak position was obtained by fitting the data in the region of the peak to a third-order polynomial. The uncertainties are presented as one standard deviation. [a]The peak position just after thermal relaxation, $\nu(0)$, was obtained by averaging the peak positions observed in the time range from 40 through 100 ps. [b]The peak position after the completion of the peak shift, $\nu(\infty)$, was obtained by averaging the peak positions observed in the time range from 1 through 50 μs for the isolated β^{SH} chains, oxyhemoglobin, and the $\alpha_2(Fe^{3+}\text{-}CN)\beta_2(Fe^{2+}\text{-}O_2)$ valency hybrid. The peak position, $\nu(\infty)$, for the isolated α^{SH} chains and the $\alpha_2(Fe^{2+}\text{-}O_2)\beta_2(Fe^{3+}\text{-}CN)$ valency hybrid was obtained by averaging the peak positions observed in the time range from 10 through 50 μs.

Table S2 Comparison of time constants of temporal changes in the ν(Fe-His) frequency following photodissociation of CO and the means of the lifetime distribution peaks extracted from the second amplitude vector, V_2

Protein	Comments	τ_1 (ns)	τ_2 (ns)	τ_3 (μs)	τ_4 (μs)
isolated α chains	Literature data	0.256 ± 0.035[a]	32.8 ± 12.4[c]	2.12 ± 0.56[c]	
	Our data[b]	0.82	21	0.69	
isolated β chains	Literature data	0.298 ± 0.042[a]	146 ± 15[c]	2.19 ± 0.40[c]	
	Our data	0.29	50	1.15	
Hb	Literature data	0.284 ± 0.038[a]	65 ± 11[d]	0.91 ± 0.29[d]	41 ± 4[d]
	Our data	0.13	26	1.12	
Hb	Literature data		20.0 ± 7.1[c,e]	0.631 ± 0.243[c,e]	17.3 ± 1.3[c,e]
	Our data		26	1.12	
α subunit in α_2(Fe)β_2(Ni)	Literature data		20.2 ± 10.1[e]	0.688 ± 0.207[e]	19.5 ± 1.6[e]
	Our data		17	0.18	
α subunit in hybrid Hb[f]	Literature data				~20[g]
β subunit in hybrid Hb[f]	Literature data			~3[g]	
	Our data			1.82	

[a]The values were taken from Mizutani and Nagai.[16]

[b]The values represent the mean of the *i*th MEM peak, τ_i ($i = \overline{1,3}$) (Table 2), in lifetime distributions extracted from the second amplitude vector, V_2.

[c]The values were taken from Yamada et al.[18] The uncertainties shown are 90% confidence levels in the curve fitting.

[d]The values were taken from Chang et al.[19]

[e]The values were taken from Yamada et al.[20] The uncertainties shown are 90% confidence levels in the curve fitting.

[f]In hybrid Hb, the native protoheme is replaced with mesoheme in either the α or β subunits.[17]

[g]The values were taken from Jones et al.[17]

Table S3 List of model parameters

Parameter	Description	Comments
β	stretched exponential parameter	constrain $0 < \beta < 1$
l	tertiary equilibrium constant	constrain $l > 1$
$k(r^* \to r)$	tertiary transition rate	
$k_{gem}(r^*)$	rate constant for the geminate O_2 rebinding to protein in the r^* tertiary conformation	constrain 1.2 $k_{gem}(r) < k_{gem}(r^*)$
$k_{gem}(r)$	rate constant for the geminate O_2 rebinding to protein in the r tertiary conformation	constrain 1.2 $k_{gem}(r) < k_{gem}(r^*)$
k_{BS}	rate constant for O_2 escape from protein	assumed independent of protein state
k_{SB}	rate constant for O_2 entry into protein	assumed independent of protein state
k_{BC}	rate constant for O_2 migration from the primary to the secondary docking site(s)	assumed independent of protein state
k_{CB}	rate constant for O_2 migration from the secondary to the primary docking site	assumed independent of protein state

Table S4 Maxima in the difference spectra between unliganded and O_2-liganded ferrous subunits in both the r^* and r tertiary structure

Protein	$\nu(r^*)$ (cm^{-1})	$\nu(r)$ (cm^{-1})	$\Delta\nu = \nu(r) - \nu(r^*)$ (cm^{-1})
αO_2	23086[a] (23103)	23187 (23170)	101 (67)
$\alpha_2(Fe^{2+}\text{-}O_2)\beta_2(Fe^{3+}\text{-}CN)$	23050 (23059)	23075 (23075)	25 (16)
$\alpha_2(Fe^{3+}\text{-}CN)\beta_2(Fe^{2+}\text{-}O_2)$	22900 (22917)	23075 (23067)	175 (150)

[a] The data obtained by the extended model are given without parenthesis, while those obtained by the basic model are presented in parenthesis. $\Delta\nu$ denotes spectral shift upon conformational relaxation.

Supplemental References

1 E. Bucci and C. Fronticelli, *J. Biol. Chem.*, 1965**,** **240**, 551–552.

2 K. M. Parkhurst and L. J. Parkhurst, *Int. J. Biochem.*, 1992**,** **24**, 993–998.

3 M. Brunori, G. Amiconi, E. Antonini and J. Wyman, *J. Mol. Biol.*, 1970, **49**, 461–471.

4 G. M. Greetham, P. Burgos, Q. Cao, I. P. Clark, P. S. Codd, R. C. Farrow, M. W. George, M. Kogimtzis, P. Matousek, A. W. Parker, M. R. Pollard, D. A. Robinson, Z.-J. Xin and M. Towrie, *Appl. Spectrosc.*, 2010**,** **64**, 1311–1319.

5 G. M. Greetham, D. Sole, I. P. Clark, A. W. Parker, M. R. Pollard and M. Towrie, *Rev. Sci. Instrum.*, 2012**,** **83**, 103107-1–5.

6 E. R. Henry and J. Hofrichter, *Methods Enzymol.*, 1992, **210**, 129–192.

7 P. J. Steinbach, K. Chu, H. Frauenfelder, J. B. Johnson, D. C. Lamb, G. U. Nienhaus, T. B. Sauke and R. D. Young, *Biophys. J.*, 1992, **61**, 235–245.

8 P. S. Steinbach, R. Ionescu and C. R. Matthews, *Biophys. J.*, 2002, **82**, 2244–2255.

9 P. J. Steinbach, *J. Chem. Inf. Comput. Sci.*, 2002, **42**, 1476–1478.

10 D. Lavalette, C. Tetreau, J.-C. Brochon, A. Livesey, *Eur. J. Biochem.*, 1991, **196**, 591–598.

11 T. J. Cornwell, K. F. Evans, *Astron. Astrophys*. 1985, **143**, 77–83.

12 J. Skilling, R. K. Bryan, *Mon. Not. R. Astr. Soc*. 1984, **211**, 111–124.

13 W. H. Press, S. A. Teukolsky, W. T. Vetterling and B. P. Flannery, *Numerical Recipes in Fortran 77: The Art of Scientific Computing*. Second Edition. Cambridge University Press: Cambridge, 1992.

14 N. Metropolis, A. W. Rosenbluth, M. N. Rosenbluth and A. H. Teller, *J. Chem. Phys.*, 1953, **21**, 1087–1092.

15 D. W. Marquardt, *J. Soc. Indust. Appl. Math.*, 1963, **11**, 431–441.

16 Y. Mizutani and M. Nagai, *Chem. Phys.*, 2012, **396**, 45–52.

17 E. M. Jones, E. Monza, G. Balakrishnan, G. C. Blouin, P. J. Mak, Q. Zhu, J. R. Kincaid, V. Guallar and T. G. Spiro, *J. Am. Chem. Soc.*, 2014, **136**, 10325–10339.

18 K. Yamada, H. Ishikawa and Y. Mizutani, *J. Phys. Chem. B*, 2012, **116**, 1992–1998.

19 S. Chang, M. Mizuno, H. Ishikawa and Y. Mizutani, *Chem. Phys.*, 2016, **469–470**, 31–37.

20 K. Yamada, H. Ishikawa, M. Mizuno, N. Shibayama and Y. Mizutani, *J. Phys. Chem. B*, 2013, **117**, 12461–12468.
